# Terazosin Attenuates Neuronal Pyroptosis by Regulating the Mitochondrial ROS/NLRP3 Inflammasome Axis Through Mitophagy in Cerebral Ischemia–Reperfusion Injury

**DOI:** 10.1002/kjm2.70242

**Published:** 2026-06-09

**Authors:** Wei Wang, Xin‐Yue Han, Li‐An Huang, Yu‐Sheng Zhang, Heng Meng, An‐Ding Xu

**Affiliations:** ^1^ Jinan University Guangzhou Guangdong China; ^2^ Department of Neurology, Neuromedical Center The University of Hong Kong‐Shenzhen Hospital Shenzhen Guangdong China; ^3^ Department of Obstetrics and Gynecology Shenzhen University General Hospital Shenzhen Guangdong China; ^4^ Department of Neurology The First Affiliated Hospital of Jinan University Guangzhou Guangdong China

**Keywords:** cerebral ischemia–reperfusion injury, mitophagy, pyroptosis, reactive oxygen species‐NOD‐like receptor protein 3, terazosin

## Abstract

Cerebral ischemia–reperfusion injury (CI/RI) is a major cause of secondary neuronal damage following ischemic stroke. This study investigated whether terazosin (TZ) exerts neuroprotective effects by regulating mitophagy and the reactive oxygen species (ROS)/NOD‐like receptor protein 3 (NLRP3) inflammasome axis. A mouse model of CI/RI was established and treated with TZ alone or in combination with the mitophagy inhibitor Mdivi‐1 or the NLRP3 activator nigericin. Neurological function, infarct volume, histopathological changes in the hippocampal CA1 region, mitophagy (Mito‐Tracker^+^LC3B^+^), and neuronal pyroptosis (NeuN^+^GSDMD‐N^+^) were evaluated. In parallel, an oxygen–glucose deprivation/reperfusion (OGD/R) model was established in HT‐22 cells to assess cell viability, cytotoxicity, mitochondrial membrane potential, ROS production, inflammatory cytokine release, and expression of autophagy‐ and pyroptosis‐related proteins. The CI/RI exhibited was characterized by worsened neurological deficits, increased infarct volume, enhanced neuronal pyroptosis, and elevated interleukin‐1β (IL‐1β), IL‐18, ROS, p62, and pyroptosis proteins, as well as prominent vacuolation and edema in the hippocampal CA1 region. These changes were accompanied by reduced mitophagy and decreased expression of LC3B II/I and Beclin‐1. TZ treatment markedly ameliorated these abnormalities. Mechanistic analyses showed that TZ inhibited OGD/R‐induced neuronal pyroptosis by promoting mitophagy, which reduced ROS accumulation and subsequently suppressed NLRP3 inflammasome activation. Importantly, blockade of mitophagy or activation of NLRP3 weakened the protective effects of TZ. Collectively, these findings indicate that TZ mitigates CI/RI‐induced neuronal injury by enhancing mitophagy and inhibiting ROS/NLRP3‐dependent pyroptosis.

## 1 Introduction

Cerebral ischemia–reperfusion injury (CI/RI) is a major pathological consequence of ischemic stroke and a critical determinant of neurological outcome [1]. Increasing evidence indicates that diverse forms of regulated cell death, including apoptosis, autophagy, necroptosis, necrosis, pyroptosis, and ferroptosis, are involved in the progression of CI/RI [2]. Elucidating the molecular basis of these processes is therefore essential for identifying effective therapeutic strategies to improve outcomes in patients after cerebral ischemic stroke.

Mitophagy serves as a crucial mitochondrial quality‐control mechanism by selectively removing dysfunctional or damaged mitochondria through the coordinated recruitment of mitophagy‐related proteins [3]. In the setting of CI/RI, appropriate activation of mitophagy can preserve mitochondrial homeostasis by eliminating injured mitochondria. However, excessive or dysregulated mitophagy may instead exacerbate neuronal damage [4]. Importantly, damaged mitochondria are a major source of reactive oxygen species (ROS), which can activate the NOD‐like receptor protein 3 (NLRP3) inflammasome and initiate downstream inflammatory injury [5]. This pathway ultimately promotes pyroptosis and amplifies tissue damage, suggesting that modulation of mitophagy and the ROS/NLRP3 axis may offer a promising therapeutic avenue in CI/RI.

Pyroptosis is a highly inflammatory form of programmed cell death characterized by cell swelling, membrane rupture, and release of pro‐inflammatory mediators, distinguishing it from apoptosis and necrosis [6]. The NLRP3 inflammasome has emerged as a key mediator of pyroptosis in CI/RI [7]. During cerebral ischemia, deprivation of oxygen and nutrients results in mitochondrial dysfunction, ATP depletion, and excessive ROS generation [8]. Upon reperfusion, restoration of oxygen paradoxically triggers a burst of ROS, which further intensifies oxidative stress and activates the NLRP3 inflammasome, thereby promoting pyroptosis and aggravating neuronal injury [9]. Accordingly, inhibition of ROS accumulation and NLRP3 inflammasome activation may represent an effective strategy for limiting pyroptosis during CI/RI.

Terazosin (TZ), a classic α1‐adrenergic receptor (AR) antagonist, has been widely used in the treatment of hypertension and benign prostatic hyperplasia [10]. More recently, TZ has attracted attention for its potential neuroprotective properties, including an association with a reduced risk of neurodegenerative diseases [11, 12]. TZ has been reported to exert anti‐inflammatory effects through inhibition of the nuclear factor‐kappa B (NF‐κB)‐gasdermin D (GSDMD) signaling pathway [13]. In addition, emerging evidence suggests that TZ may improve mitochondrial integrity, restore mitochondrial function, and promote autophagic clearance of damaged mitochondria [14]. These observations raise the possibility that TZ may alleviate neurological injury by regulating mitochondrial homeostasis, oxidative stress, and inflammatory signaling. However, whether TZ exerts protective effects in CI/RI, and the mechanisms involved, remain unknown. Therefore, this study aimed to determine whether TZ attenuates CI/RI by regulating mitochondrial function and the ROS/NLRP3 signaling pathway to suppress inflammation and neuronal pyroptosis, thereby providing a potential theoretical basis for therapeutic intervention.

## 2 Materials and Methods

### 2.1 Ethical Approval

All animal procedures conformed to the guidelines published by the National Institutes of Health (NIH) for the Care and Use of Laboratory Animals (revised in 1978). The experimental protocol was approved by the Animal Care and Use Committee of our hospital (Approval No. 2024‐053; date: 2024‐05‐17).

### 2.2 Experimental Animals

A total of 198 male C57BL/6 mice (8 weeks old, 25–30 g) were obtained from Agna Biopharmaceutical Co. Ltd. (Guangzhou, China). Prior to experimentation, the mice underwent a one‐week acclimatization period under controlled laboratory conditions (temperature: 20°C–24°C, humidity: 40%–60%, 12‐h light/dark cycle) with ad libitum access to food and water.

Humane endpoint criteria were established to minimize animal distress, including: (1) body weight loss ≥ 20% of the initial body weight; (2) complete anorexia for 24 h or food intake < 50% of normal requirements for 72 h; (3) severe dehydration or persistent diarrhea/vomiting for more than 24 h; (4) respiratory distress, abnormal tremors, or pain‐related behaviors (e.g., limb retraction, or bruxism).

Animals meeting any of these criteria were immediately euthanized by intraperitoneal injection of sodium pentobarbital (150 mg/kg, P3761, Sigma‐Aldrich, St Louis, MO, USA).

### 2.3 Animal Grouping and Treatment

The cerebral ischemia–reperfusion injury (CI/RI) model was established using the middle cerebral artery occlusion/reperfusion (MCAO/R) method as previously described [15]: Briefly, mice were anesthetized with 2% isoflurane and maintained under 0.5%–1% isoflurane during surgery. The animals were placed in a supine position, and a midline cervical incision was made to expose the right common carotid artery, internal carotid artery (ICA), and external carotid artery (ECA). A heparinized intravascular monofilament (diameter: 0.22 ± 0.02 mm) with a rounded tip was inserted through the ECA into the ICA and advanced to occlude the origin of the middle cerebral artery. After 1 h of occlusion, the filament was carefully withdrawn to allow reperfusion. Mice in the sham group underwent the same surgical procedures except for the insertion of the monofilament. During the entire surgical procedure, body temperature was maintained at 36.5°C ± 0.5°C using a heat lamp until full recovery from anesthesia.

After successful model establishment, mice were randomly divided into the following groups (*n* = 18 per group): Sham group: mice underwent sham surgery without filament insertion; MCAO/R group: mice subjected to middle cerebral artery occlusion/reperfusion (MCAO/R) modeling; MCAO/*R* + TZ group: mice received intraperitoneal injections of terazosin (TZ; HY‐B0371, MedChemExpress, Monmouth Junction, NJ, USA) at doses of 0.1, 1, 10, or 100 μg/kg 30 min before occlusion [12]; MCAO/*R* + TZ + PE group: mice received intraperitoneal injections of 10 μg/kg TZ and 5 mg/kg phenylephrine (PE, an α1‐AR agonist; Sigma) at the onset of reperfusion [16]; MCAO/*R* + TZ + M group: mice received intraperitoneal injections of 10 μg/kg TZ and 3 mg/kg Mdivi‐1 (a mitophagy inhibitor; HY‐15886, MCE) at the onset of reperfusion [17]; MCAO/*R* + TZ + V1 group: mice were administered 10 μg/kg TZ together with the corresponding vehicle for Mdivi‐1 (10% dimethyl sulfoxide [DMSO] + 90% corn oil); MCAO/*R* + TZ + *N* group: mice received intraperitoneal injections of 10 μg/kg TZ and 4 mg/kg Nigericin (an NLRP3 activator; HY‐160638, MCE) immediately after MCAO surgery [7]; MCAO/*R* + TZ + V2 group: mice were administered 10 μg/kg TZ together with the vehicle control for nigericin (10% ethanol + 90% corn oil).

After 24 h of reperfusion, neurological deficit scoring was assessed in all mice. Subsequently, animals were euthanized by intraperitoneal injections of sodium pentobarbital (150 mg/kg). Brain tissues were then collected for subsequent analyses. Specifically, six randomly selected mice from each group were used for 2,3,5‐triphenyltetrazolium chloride (TTC) staining to evaluate cerebral infarct volume. The hippocampal CA1 region of the right hemisphere from another six mice was fixed in 4% paraformaldehyde, embedded in paraffin, and processed for hematoxylin and eosin (H&E) staining and immunofluorescence analysis. The remaining six mice had the right hippocampal CA1 region homogenized for western blot analysis, flow cytometry, and enzyme‐linked immunosorbent assay (ELISA).

Effect size analysis indicated that the effect sizes (*η* ^2^) in this study were greater than 0.16. Post hoc power analysis based on these effect sizes yielded a statistical power of 0.99 (Figure S1).

### 2.4 Assessment of Neurological Deficits

Neurological function was evaluated 24 h after reperfusion using the modified neurological severity score (mNSS). The mNSS is a composite scoring system that includes motor tests, tail suspension tests, placing tests, proprioception assessments, balance beam tests, and evaluations of reflex loss and abnormal movement. Scores range from 0 to 18, where 0 indicates normal neurological function, 1–6 indicates mild neurological deficits, 7–12 indicates moderate neurological deficits, and 13–18 indicates severe neurological deficits.

### 2.5 TTC Staining

Whole brains were rapidly removed and briefly rinsed with saline. Coronal brain sections were cut from anterior to posterior into five slices, each with a thickness of 2 mm. The slices were incubated in phosphate‐buffered saline (PBS) comprising 2% 2,3,5‐triphenyltetrazolium chloride (TTC; Sigma‐Aldrich) at 37°C for 30 min in the dark. After staining, the sections were fixed in 4% paraformaldehyde (Beyotime, Shanghai, China) for 2 h and photographed.

Image analyses were performed using ImageJ software (National Institutes of Health, Bethesda, MD, USA). The infarct volume of each slice was calculated by multiplying the infarct area by the slice thickness (2 mm). Infarcted brain tissue appeared white, while viable tissue was stained red.

### 2.6 Hematoxylin and Eosin (H&E) Staining

Histopathological damage in the hippocampal CA1 region was examined using hematoxylin and eosin (H&E) staining. Paraffin‐embedded tissue sections were stained using an H&E staining kit (Beyotime) according to the manufacturer's instructions. The stained sections were observed and imaged under an automated biological microscope (Leica DM6000B, Wetzlar, Germany).

The denatured cell index was calculated using the following formula:

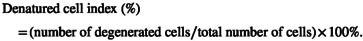




### 2.7 Immunofluorescence

To evaluate mitophagy, tissue sections from the hippocampal CA1 region were incubated with 400 nM Mito‐Tracker Green (#C1048, Beyotime) for 40 min. Sections were subsequently blocked with 1% bovine serum albumin (BSA; #735094; Amresco, Solon, OH, USA) in PBS for 1 h. The sections were then incubated with rabbit anti‐LC3B primary antibody (1:100, ab192890, Abcam, Cambridge, UK), followed by incubation with a 647‐conjugated goat anti‐rabbit IgG secondary antibody (1:200, ab150083, Abcam). Nuclei were counterstained with 5 μg/mL 4′,6‐diamidino‐2‐phenylindole (DAPI; #C1002, Beyotime) for 3 min at room temperature. Fluorescence images were acquired using a laser confocal microscope (Olympus, Tokyo, Japan).

For pyroptosis assessment, paraffin sections of hippocampal tissue were deparaffinized with xylene and rehydrated through gradient ethanol. The sections were then incubated overnight at 4°C with primary antibodies against GSDMD‐N (1:50, PA5‐115330, Thermo Fisher, Waltham, MA, USA) and NeuN (ab177487, Abcam). Subsequently, the sections were incubated with Alexa Fluor 488‐conjugated goat anti‐rabbit IgG secondary antibody (1:500, ab150077, Abcam) for 2 h at room temperature.

After washing with PBS, the sections were mounted with DAPI for nuclear counterstaining and observed using a confocal laser scanning microscope (Olympus). The number of three‐positive overlapping cells (NeuN + GSDMD‐*N* + DAPI) was quantified to determine the pyroptosis rate.

Immunofluorescence imaging was performed using an Olympus FV3000 laser confocal microscope (Tokyo, Japan). Image acquisition and analysis were conducted using Olympus proprietary software for three‐dimensional reconstruction, while fluorescence intensity and co‐localization were quantified using ImageJ (National Institutes of Health).

### 2.8 Cell Culture

Mouse hippocampal neuronal HT‐22 cells were purchased from iCell Bioscience (Shanghai, China). The cells were cultured in Dulbecco's Modified Eagle's Medium (DMEM; Gibco, Thermo Fisher Scientific, Waltham, MA, USA) supplemented with 10% fetal bovine serum (FBS; Gibco) and 1% penicillin–streptomycin (Zeye Biotechnology Co. Ltd., Shanghai, China). Cells were maintained in a humidified incubator at 37°C with 5% CO_2_.

### 2.9 Establishment of the Oxygen‐Glucose Deprivation/Reperfusion (OGD/R) Cell Model

An in vitro model of CI/RI was established using oxygen–glucose deprivation/reperfusion (OGD/R) as previously described [18]. Briefly, HT‐22 cells were incubated in serum‐free and glucose‐free DMEM (Gibco) and placed in a hypoxic chamber containing 95% N_2_ and 5% CO_2_ at 37°C for 6 h. Subsequently, the medium was replaced with complete DMEM, and the cells were returned to normoxic conditions (5% CO_2_, 37°C) for 18 h to simulate reperfusion. Cells maintained under normal culture conditions served as the control (Blank group).

### 2.10 Cell Grouping and Treatment

HT‐22 cells were randomly divided into the following seven groups: Blank group: cells cultured under normal conditions; OGD/R group: cells subjected to OGD/R treatment; OGD/*R* + TZ group: cells pretreated with 10 nM TZ [13, 19] prior to OGD/R induction; OGD/*R* + TZ + M group: cells treated with 10 nM TZ and 10 μM Mdivi‐1 (a mitophagy inhibitor) [20] for 48 h before OGD/R induction; OGD/*R* + TZ + D group: cells treated with 10 nM TZ and 10 μM DMSO prior to OGD/R induction; and OGD/*R* + TZ + *N* group: cells treated with 10 nM TZ and 10 μM nigericin (an NLRP3 inflammasome activator) [21] followed by OGD/R induction.

### 2.11 3‐[4,5‐Dimethylthiazol‐2‐Yl]‐2,5‐Diphenyl Tetrazolium Bromide (MTT) Assay

Cell viability was assessed using an MTT assay kit (BB‐4201‐250T, Zeye Biotechnology Co. Ltd.) according to the manufacturer's instructions. The optical density (OD) at 490 nm was measured using a microplate reader (Thermo Fisher Scientific).

### 2.12 Detection of Lactate Dehydrogenase (LDH) Activity

Cell injury was evaluated by measuring lactate dehydrogenase (LDH) release using an LDH Cytotoxicity Detection Kit (A020‐1‐2, Jiancheng Bioengineering Institute, Nanjing, Jiangsu, China) according to the manufacturer's instructions.

### 2.13 Detection of Mitochondrial Membrane Potential (MMP)

Changes in mitochondrial membrane potential (MMP) were detected using a JC‐1 MMP detection kit (C2003S, Beyotime). The assay was performed according to the manufacturer's protocol. After staining, cells were resuspended and observed under a fluorescence microscope (Keyence Corporation, Osaka, Japan). When MMP was high, JC‐1 formed aggregates that emitted red fluorescence, whereas decreased MMP resulted in JC‐1 monomers emitting green fluorescence.

### 2.14 Flow Cytometry

Cell pyroptosis was analyzed using propidium iodide (PI; #KGA214, KeyGEN Biotech, Nanjing, Jiangsu, China) and a FAM‐FLICA caspase 1 detection kit (#97, ImmunoChemistry Technologies LLC, Bloomington, MN, USA). According to the manufacturer's instructions, harvested cells were co‐incubated with FAM‐FLICA caspase‐1 reagent and PI. Cells positive for both PI and caspase‐1 fluorescence were analyzed using a FACSCalibur flow cytometer (Becton Dickinson, San Jose, CA, USA).

### 2.15 Elisa

The levels of interleukin‐1β (IL‐1β; PI301) and interleukin‐18 (IL‐18; PI553) in hippocampal CA1 tissue homogenates or cell culture supernatants were measured using commercial ELISA kits (Beyotime) according to the manufacturer's instructions. The optical density (OD) was measured at 450 nm using a microplate reader (Bio‐Rad Laboratories, Hercules, CA, USA).

### 2.16 Determination of Reactive Oxygen Species (ROS)

Reactive oxygen species (ROS) levels in hippocampal CA1 tissue homogenates or HT‐22 cells were determined using a ROS detection kit (S0033S, Beyotime) following the manufacturer's protocol.

### 2.17 Western Blot Analysis

Total proteins were extracted from HT‐22 cells or hippocampal CA1 tissue homogenates using radio‐immunoprecipitation assay (RIPA) lysis buffer (Beyotime) supplemented with protease inhibitors (Complete Mini, Roche, Basel, Switzerland). Protein concentrations were determined using a BCA Protein Assay Kit (Beyotime).

Equal amounts of protein (50 μg) were separated by 10% sodium dodecyl sulfate‐polyacrylamide gel electrophoresis (SDS‐PAGE) and transferred onto polyvinylidene fluoride (PVDF) membranes (Millipore, Billerica, MA, USA). The membranes were blocked with 5% skim milk in Tris Buffered Saline containing Tween‐20 (TBST, Beyotime) for 1 h at room temperature and then incubated overnight at 4°C with the following primary antibodies: rabbit anti‐NLRP3 (1:2000, ab263899, Abcam), Cleaved caspase‐1 (1:2000, ab179515, Abcam), GSDMD‐N (1:2000, 39,754, CST, Danvers, MA, USA), apoptosis‐associated spot‐like protein (ASC; 1:2000, ab307560, Abcam), LC3B (1:2000, ab192890, Abcam), Beclin‐1 (1:2000, ab207612, Abcam), p62 (1:2000, ab109012, Abcam), and β‐actin (1:10000, ab8227, Abcam).

After washing with TBST, the membranes were incubated with goat anti‐rabbit IgG (H&L) HRP‐conjugated secondary antibody (1:10,000, ab6721, Abcam) for 1 h at room temperature. Protein bands were visualized using an enhanced chemiluminescence (ECL) detection system (Seyotin, Guangzhou, Guangdong, China). Band intensities were quantified using ImageJ software (National Institutes of Health), with β‐actin used as the internal loading control.

### 2.18 Statistical Analysis

All statistical analyses and graphical representations were performed and analyzed using GraphPad Prism 9.5 (GraphPad Software Inc., San Diego, CA, USA). Data normality was assessed using the Kolmogorov–Smirnov test. Data are presented as mean ± standard deviation (SD). Comparisons among multiple groups were performed using one‐way analysis of variance (ANOVA) followed by Tukey's multiple comparison test. Statistical metrics, including *p*‐values, effect sizes (*η* ^2^), and confidence intervals, were calculated where applicable. A *p* value < 0.05 was considered statistically significant.

## 3 Results

### 3.1 **TZ** Exerted Dose‐Dependent Protective Effects Against CI/RI in Mice Within an Optimal Dose Range

**FIGURE 1:**
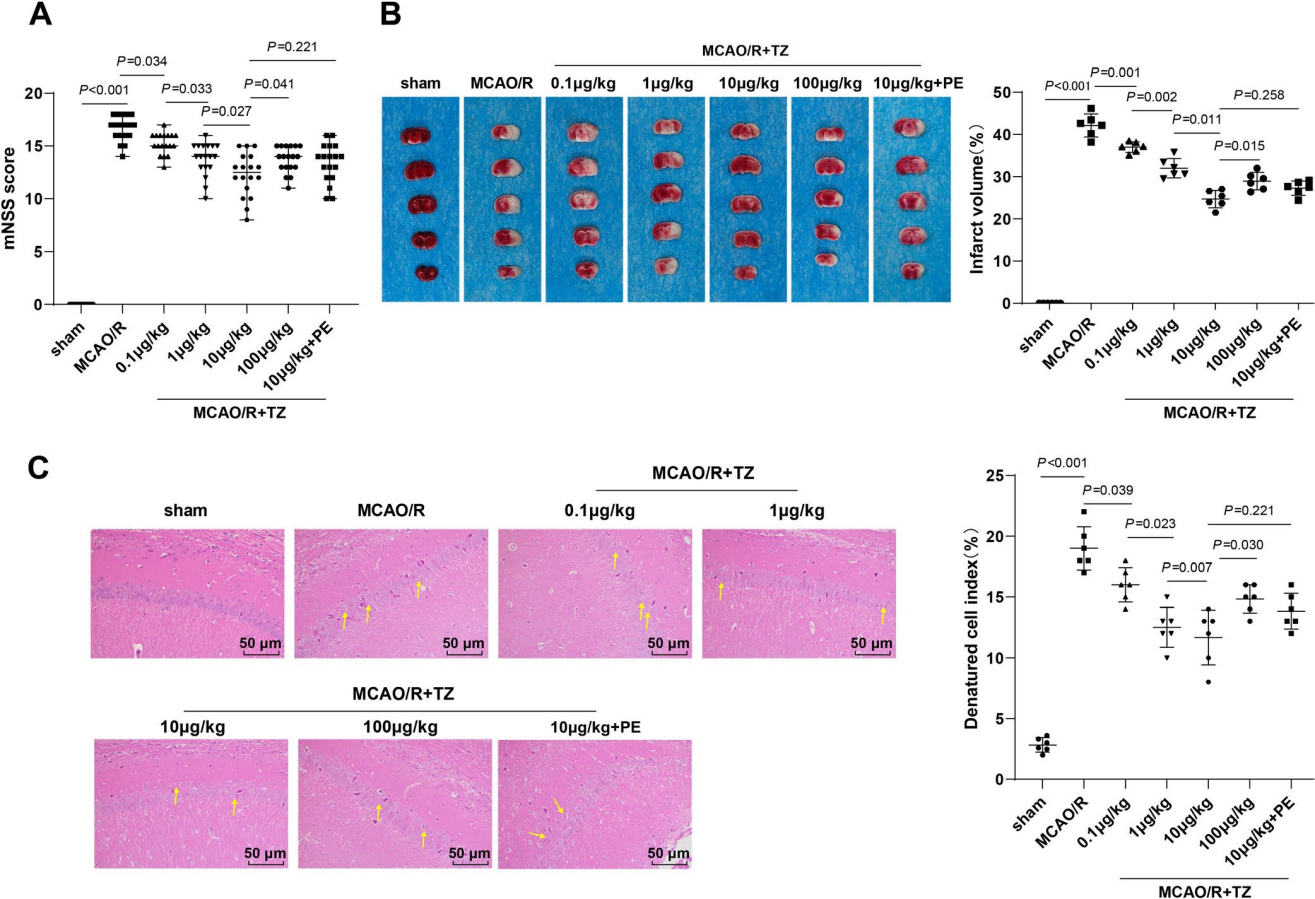
TZ alleviated CI/RI in mice in a dose‐dependent manner within a certain range. (A) mNSS scores were used to assess neurological deficits (*η* ^2^ = 0.934, 95% CI 11.211–13.090), *n* = 18. (B) Representative images of coronal brain sections stained with TTC and quantitative analysis of cerebral infarction volume (*η* ^2^ = 0.981, 95% CI 23.462–31.414), *n* = 6. (C) H&E staining showing pathological tissue damage in the hippocampal CA1 region (*η* ^2^ = 0.917, 95% CI 11.405–14.499), *n* = 6. Data comparisons among multiple groups were analyzed using one‐way ANOVA, followed by Tukey's multiple comparison test. *p* < 0.05 was considered statistically significant. TZ, terazosin; PE, phenylephrine.

To investigate the protective effects of TZ in CI/RI, a middle cerebral artery occlusion/reperfusion (MCAO/R) mouse model was first established [22]. As shown in Figure 1A, mice in the MCAO/R group exhibited significantly higher modified neurological severity score (mNSS) values compared with those in the sham group, indicating the presence of neurological deficits following ischemia–reperfusion injury.

TTC staining further revealed a markedly larger infarct area in the brain tissues of the MCAO/R group compared with the sham group (Figure 1B). Consistent with these findings, H&E staining showed that neurons in the hippocampal CA1 region of sham mice displayed normal morphology, characterized by intact cellular structure and clearly defined nuclei and cytoplasm. In contrast, neurons in the MCAO/R group exhibited severe neuronal structural damage, including cytoplasmic vacuolation, irregular nuclear morphology, and a significantly increased denatured cell index (Figure 1C). These results confirmed the successful establishment of the MCAO/R‐induced CI/RI mouse model.

Next, mice were treated with different doses of TZ (0.1, 1, 10, and 100 μg/kg). TZ treatment significantly alleviated brain injury, as evidenced by reduced mNSS scores, decreased infarct volume, and a lower denatured cell index. Histological analysis also showed reduced neuronal damage, nuclear fragmentation, and cellular vacuolation. The protective effects of TZ were dose‐dependent within the range of 0.1–10 μg/kg. However, when the TZ dose reached 100 μg/kg, the neuroprotective effects were attenuated (Figure 1A–C). Therefore, a dose of 10 μg/kg TZ was selected for subsequent experiments.

Since TZ is a classic α1‐AR antagonist, a rescue experiment was performed to determine whether its neuroprotective effects were mediated through α1‐AR antagonism. Mice were treated with TZ (10 μg/kg) in combination with the α1‐AR agonist, PE. The results showed no significant differences between the MCAO/*R* + TZ group and the MCAO/*R* + TZ + PE group (Figure 1A–C). These findings indicate that TZ effectively alleviates CI/RI in mice in a dose‐dependent manner within a specific concentration range.

### 3.2 TZ Enhanced Mitophagy and Inhibited Neuronal Pyroptosis in the Hippocampal CA1 Region of CI/RI Mice

To determine whether TZ regulates mitophagy in CI/RI, mitophagy levels in the hippocampal CA1 region were evaluated by immunofluorescence staining.

**FIGURE 2:**
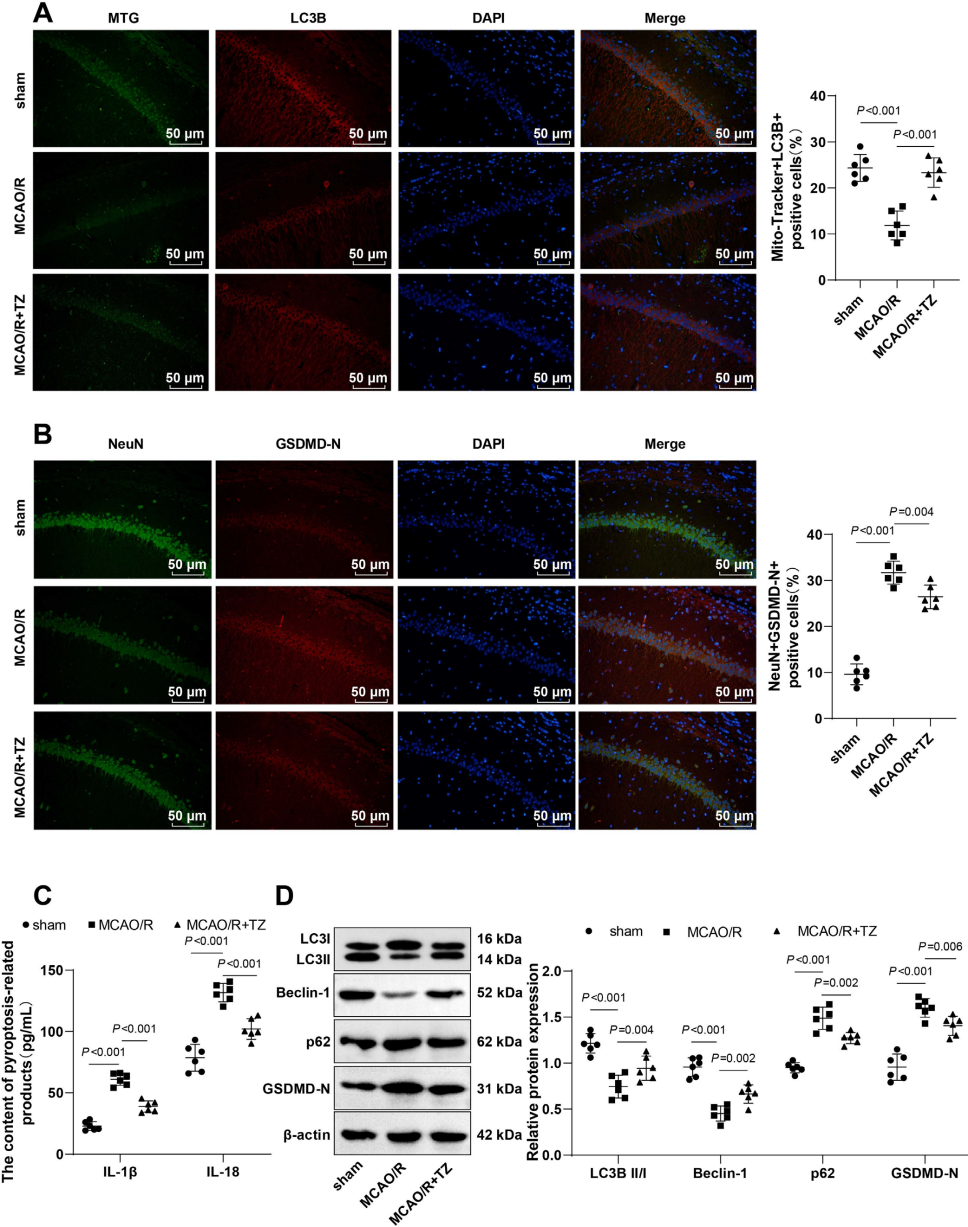
TZ inhibited neuronal pyroptosis in CI/RI mice by activating mitophagy in the CA1 region of the hippocampus. (A) Immunofluorescence detection of mitophagy (Mito Tracker^+^LC3B^+^) (*η* ^2^ = 0.801, 95% CI 16.591–23.075). (B) Immunofluorescence detection of neuronal pyroptosis (NeuN^+^GSDMD‐N^+^) (*η* ^2^ = 0.947, 95% CI 17.624–27.533), GSDMD‐N (green), NeuN (red), and DAPI (blue). (C) ELISA to determine the levels of inflammatory factors IL‐1β (*η* ^2^ = 0.937, 95% CI 32.714–49.260) and IL‐18 (*η* ^2^ = 0.873, 95% CI 92.226–116.009). (D) Western blot detection of the levels of autophagic proteins (LC3B II/I [*η* ^2^ = 0.755, 95% CI 0.855–1.082], Beclin‐1 [*η* ^2^ = 0.851, 95% CI 0.576–0.805], p62 [*η* ^2^ = 0.891, 95% CI 1.117–1.357]) and the pyroptosis protein (GSDMD‐N) (*η* ^2^ = 0.863, 95% CI 1.172–1.469). *n* = 6. One‐way ANOVA was implemented for multi‐group comparisons. Tukey's multiple comparison test was performed afterward. *p* < 0.05 was considered statistically significant. TZ, terazosin.

As shown in Figure 2A, mitophagy (Mito‐Tracker^+^LC3B^+^) was significantly reduced in the MCAO/R group compared with the sham group. However, mitochondrial LC3B expression was markedly increased in the MCAO/*R* + TZ group compared with the MCAO/R group (Figure 2A), indicating enhanced mitophagy following TZ treatment.

Consistently, Western blot analysis showed that the levels of autophagy‐related proteins LC3B II/I and Beclin‐1 were significantly decreased, while p62 protein expression was increased in the hippocampal CA1 region of the MCAO/R group compared with sham mice. In contrast, TZ treatment significantly increased LC3B II/I and Beclin‐1 levels and decreased p62 protein expression compared with the MCAO/R group (Figure 2D), further suggesting that TZ promoted mitophagy in CI/RI mice.

To evaluate neuronal pyroptosis, immunofluorescence staining for NeuN and GSDMD‐N was performed in the hippocampal CA1 region. The results showed a marked increase in NeuN^+^GSDMD‐*N* ^+^ cells in the MCAO/R group compared with the sham group, indicating enhanced neuronal pyroptosis following ischemia–reperfusion injury. In contrast, TZ treatment significantly reduced the number of NeuN^+^GSDMD‐*N* ^+^ cells in the hippocampal CA1 region compared with the MCAO/R group (Figure 2B).

ELISA results further demonstrated that IL‐1β and IL‐18 levels were significantly elevated in the hippocampal CA1 region of the MCAO/R group compared with sham mice. However, these inflammatory cytokines were significantly reduced following TZ treatment (Figure 2C). In addition, Western blot analysis showed that GSDMD‐N protein expression was markedly increased in the MCAO/R group, but significantly decreased in the MCAO/*R* + TZ group (Figure 2D).

Collectively, these results indicate that TZ enhances mitophagy in the hippocampal CA1 region of CI/RI mice and concurrently suppresses neuronal pyroptosis.

### 3.3 Mitophagy Inhibition in the Hippocampal CA1 Region Enhanced Neuronal Pyroptosis and Partially Abrogated the Protective Effects of TZ Against CI/RI in Mice

**FIGURE 3:**
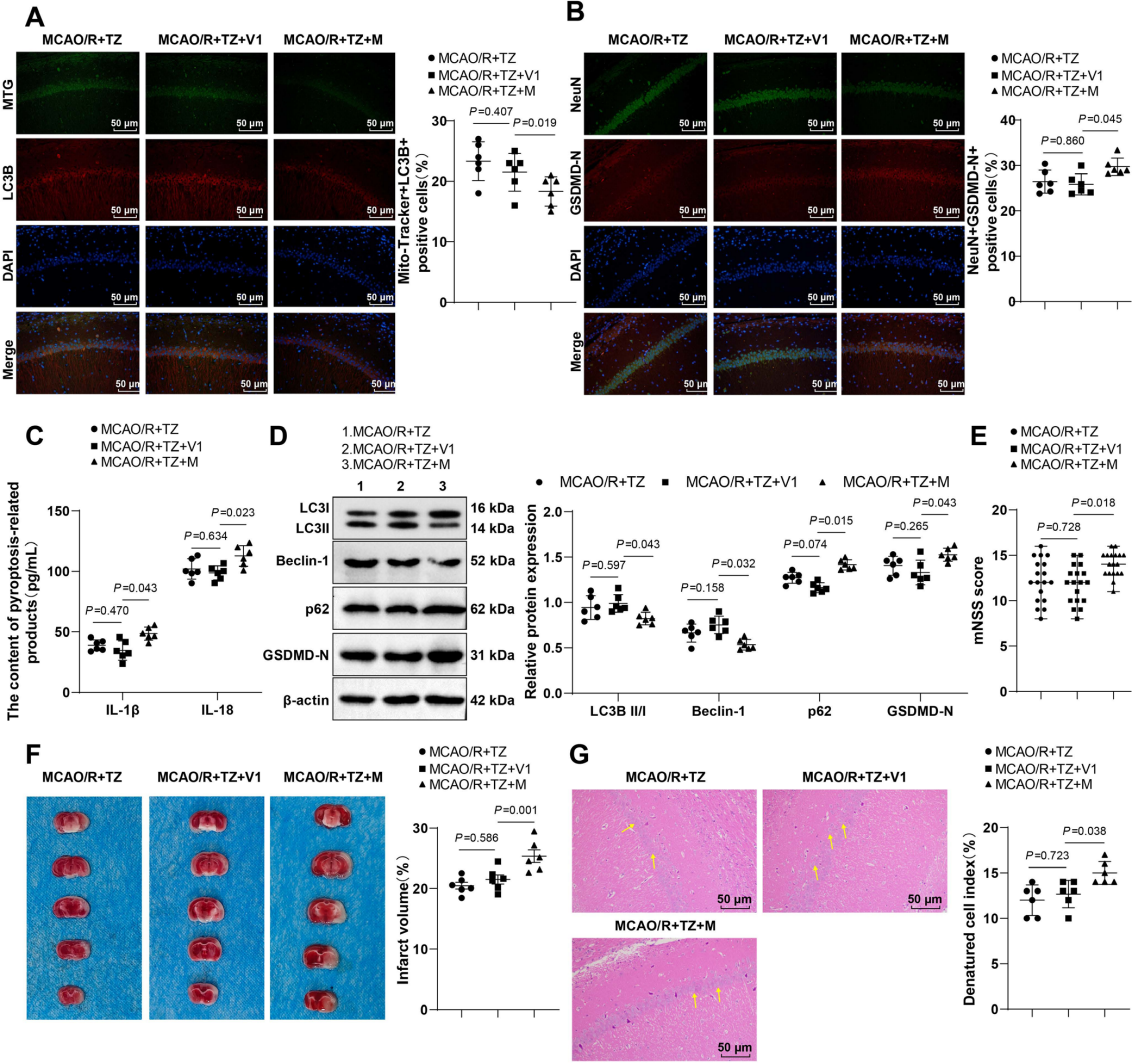
Inhibition of mitophagy in the hippocampal CA1 region enhanced neuronal pyroptosis and partially reversed the improvement effect of TZ in CI/RI mice. (A) Immunofluorescence to assess mitochondria (Mito‐Tracker^+^LC3B^+^) (*η* ^2^ = 0.371, 95% CI 19.320–22.791), *n* = 6. (B) Immunofluorescence to evaluate neuronal pyroptosis (NeuN^+^GSDMD‐N^+^) (*η* ^2^ = 0.402, 95% CI 25.954–28.719), GSDMD‐N (green), NeuN (red), and DAPI (blue), *n* = 6. (C) ELISA to test IL‐1β (*η* ^2^ = 0.519, 95% CI 36.555–44.820) and IL‐18 levels (*η* ^2^ = 0.417, 95% CI 99.774–109.250), *n* = 6. (D) Western blot to test levels of autophagic proteins (LC3B II/I [*η* ^2^ = 0.364, 95% CI 0.860–0.979], Beclin‐1 [*η* ^2^ = 0.559, 95% CI 0.589–0.712], p62 [*η* ^2^ = 0.795, 95% CI 1.224–1.342]) and the pyroptosis protein (GSDMD‐N) [*η* ^2^ = 0.408, 95% CI 1.356–1.485], *n* = 6. (E) mNSS scores were utilized to test neurological deficits (*η* ^2^ = 0.167, 95% CI 12.167–13.352), *n* = 18. (F) Representative images of coronal brain sections stained with TTC and quantitative analysis of cerebral infarction volume (*η* ^2^ = 0.638, 95% CI 21.046–23.737), *n* = 6. (G) H&E staining displaying pathological tissue damage in the hippocampal CA1 region (*η* ^2^ = 0.472, 95% CI 12.264–14.180), *n* = 6. Multi‐group data comparisons were conducted using one‐way ANOVA, followed by Tukey's multiple comparison test. *p* < 0.05 was considered statistically significant. M, Mdivi‐1; TZ, terazosin; V, Vehicle.

To further investigate whether mitophagy mediates the neuroprotective effects of TZ, CI/RI mice were co‐treated with TZ and the mitophagy inhibitor Mdivi‐1. Compared with the MCAO/*R* + TZ + V1 group, mice in the MCAO/*R* + TZ + M group exhibited significantly reduced mitophagy (Mito‐Tracker^+^LC3B^+^), accompanied by decreased expression of LC3B II/I and Beclin‐1 and increased p62 protein levels (Figure 3A,D). These findings indicate effective inhibition of mitophagy following Mdivi‐1 treatment.

Furthermore, compared with the MCAO/*R* + TZ + V1 group, the MCAO/*R* + TZ + M group displayed significantly increased neuronal pyroptosis (NeuN^+^GSDMD‐N^+^) in the hippocampal CA1 region. Consistently, IL‐1β and IL‐18 levels and GSDMD‐N protein expression were also significantly elevated (Figure 3B–D).

Importantly, inhibition of mitophagy partially reversed the protective effects of TZ against CI/RI, as evidenced by aggravated neurological deficits, increased infarct volume, and worsened histopathological injury (Figure 3E–G). These results suggest that suppression of mitophagy promotes neuronal pyroptosis and partially abolishes the neuroprotective effects of TZ in CI/RI mice.

### 3.4 TZ Inhibited OGD/R‐Induced Pyroptosis in HT‐22 Cells Through Activation of Mitophagy

**FIGURE 4:**
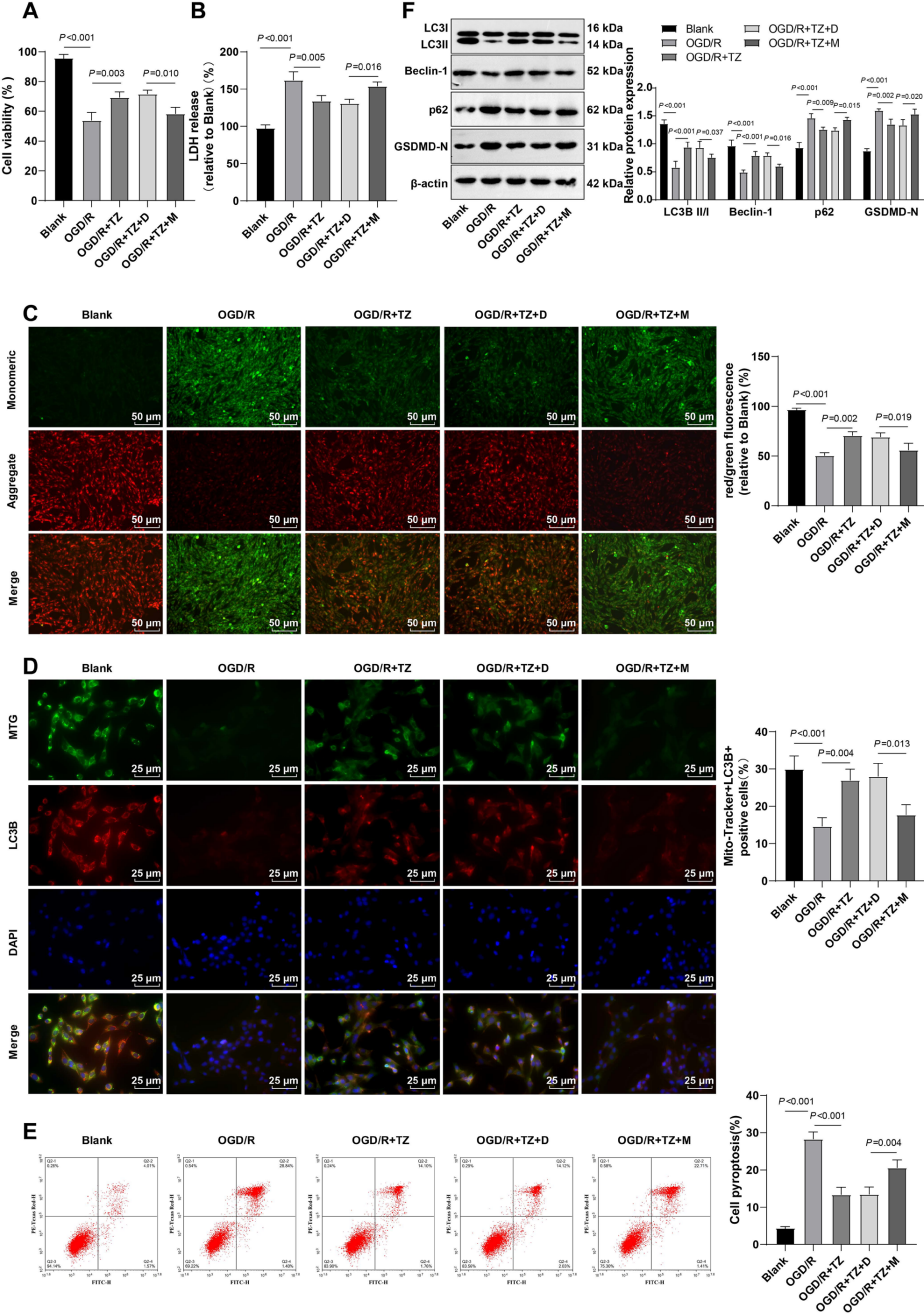
TZ inhibited OGD/R‐induced pyroptosis in HT‐22 cells by activating mitophagy. (A) MTT assay to assess cell viability (*η* ^2^ = 0.958, 95% CI 61.298–78.352). (B) LDH assay to assess cell death (*η* ^2^ = 0.938, 95% CI 122.588–149.062). (C) JC‐1 probe method to detect changes in MMP (*η* ^2^ = 0.957, 95% CI 59.301–78.089). When MMP was high, JC‐1 aggregated to emit red fluorescence; when MMP was low (damaged), JC‐1 emitted green fluorescence in the form of monomer. (D) Immunofluorescence assessment of mitophagy (Mito‐Tracker^+^LC3B^+^) (*η* ^2^ = 0.981, 95% CI 19.719–27.267). (E) Flow cytometry detection of pyroptosis (PI^+^Caspase‐1^+^) (*η* ^2^ = 0.968, 95% CI 11.380–20.64). (F) Western blot analysis of the levels of autophagic proteins (LC3B II/I [*η* ^2^ = 0.924, 95% CI 0.757–1.069], Beclin‐1 [*η* ^2^ = 0.906, 95% CI 0.630–0.828], p62 [*η* ^2^ = 0.934, 95% CI 1.152–1.377]) and the pyroptosis protein (GSDMD‐N) (*η* ^2^ = 0.940, 95% CI 1.186–1.484). Cell experiments were performed independently three times. Data were expressed as mean ± SD. Data comparisons among multiple groups were performed using one‐way ANOVA, followed by Tukey's multiple comparison test. *p* < 0.05 was considered statistically significant. D, DMSO; M, Mdivi‐1; TZ, terazosin.

To further explore the mechanism of TZ in vitro, an OGD/R‐induced CI/RI model was established using mouse hippocampal HT‐22 neurons. The MTT assay showed that cell viability was significantly reduced in the OGD/R group compared with the Blank group, whereas TZ treatment significantly improved cell viability (Figure 4A). Consistently, LDH assay results revealed increased cell death following OGD/R treatment, which was significantly attenuated by TZ (Figure 4B).

MMP analysis demonstrated that OGD/R treatment caused a significant reduction in MMP compared with the Blank group, indicating mitochondrial dysfunction. However, TZ treatment partially restored MMP levels (Figure 4C). Immunofluorescence staining further showed that mitophagy (Mito‐Tracker^+^LC3B^+^) was significantly decreased in the OGD/R group compared with the Blank group, but was markedly enhanced following TZ treatment (Figure 4C,D).

Flow cytometry analysis demonstrated that the proportion of pyroptotic cells (PI^+^/Caspase‐1^+^) was significantly increased after OGD/R treatment compared with the Blank group. TZ treatment significantly reduced the number of pyroptotic cells (Figure 4E). Consistent with these observations, Western blot showed that OGD/R treatment decreased LC3B II/I and Beclin‐1 expression while increasing p62 and GSDMD‐N protein levels. In contrast, TZ treatment reversed these changes (Figure 4F). Moreover, inhibition of mitophagy partially reversed the protective effects of TZ against OGD/R‐induced pyroptosis in HT‐22 cells (Figure 4A–F).

Collectively, these findings suggest that TZ suppresses OGD/R‐induced pyroptosis in HT‐22 cells by activating mitophagy.

### 3.5 TZ Attenuated OGD/R‐Induced NLRP3 Inflammasome Activation by Enhancing Mitophagy and Reducing ROS Accumulation in HT‐22 Cells

We next evaluated whether TZ regulates oxidative stress and NLRP3 inflammasome activation during OGD/R injury. Compared with the Blank cells, OGD/R treatment significantly increased intracellular ROS levels. In addition, the protein expression of NLRP3, ASC, and cleaved caspase‐1 was markedly elevated, accompanied by increased secretion of IL‐1β and IL‐18 in the cell supernatant.

**FIGURE 5:**
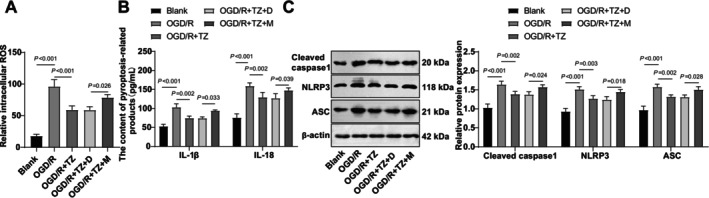
TZ decreased ROS accumulation by activating mitophagy, thus suppressing NLRP3 inflammasome activation in OGD/R‐induced HT‐22 cells. (A) Determination of ROS levels by kits (*η* ^2^ = 0.959, 95% CI 46.924–77.426). (B) ELISA to measure the levels of IL‐1β (*η* ^2^ = 0.938, 95% CI 69.512–90.218) and IL‐18 (*η* ^2^ = 0.923, 95% CI 110.803–144.838). (C) Measurement of NLRP3 (*η* ^2^ = 0.915, 95% CI 1.160–1.402), ASC (*η* ^2^ = 0.923, 95% CI 1.210–1.462), and Cleaved caspase‐1 (*η* ^2^ = 0.915, 95% CI 1.275–1.528) protein levels by western blot. Cell experiments were repeatedly implemented thrice, and the data were expressed as mean ± SD. One‐way ANOVA was utilized for multi‐group comparisons, followed by Tukey's multiple comparison test. *p* < 0.05 was considered statistically significant. D, DMSO; M, Mdivi‐1; TZ, terazosin.

TZ treatment significantly reduced ROS accumulation and downregulated the expression of NLRP3, ASC, and cleaved caspase‐1. TZ also decreased IL‐1β and IL‐18 levels. Importantly, inhibition of mitophagy partially abolished the regulatory effects of TZ on ROS production and NLRP3 inflammasome activation (Figure 5A–C).

These results indicate that TZ activates mitophagy to reduce ROS accumulation, thereby suppressing NLRP3 inflammasome activation in OGD/R‐stimulated HT‐22 cells.

### 3.6 NLRP3 Inflammasome Activation Partially Counteracted the Protective Effect of TZ on OGD/R‐Induced Pyroptosis in HT‐22 Cells

**FIGURE 6:**
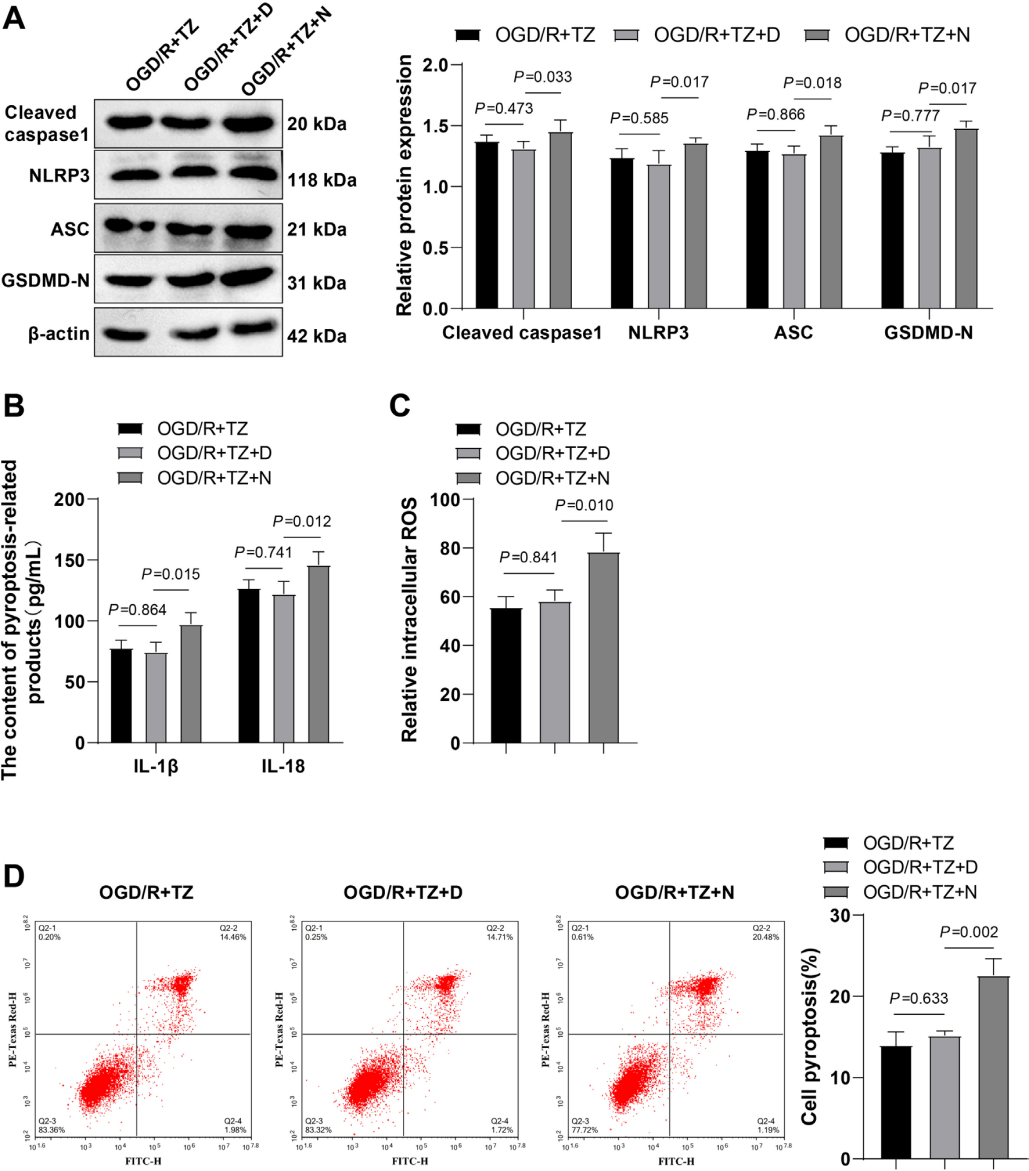
Activation of NLRP3 partly abolished the amelioration of TZ on OGD/R‐induced pyroptosis in HT‐22 cells. (A) Detection of NLRP3 (*η* ^2^ = 0.582, 95% CI 1.218–1.358), ASC (*η* ^2^ = 0.644, 95% CI 1.281–1.406), Cleaved caspase‐1 (*η* ^2^ = 0.534, 95% CI 1.317–1.448), and GSDMD‐N (*η* ^2^ = 0.689, 95% CI 1.262–1.403) protein levels by western blot. (B) Determination of IL‐1β (*η* ^2^ = 0.726, 95% CI 74.326–93.143) and IL‐18 (*η* ^2^ = 0.649, 95% CI 122.672–143.822) levels by ELISA. (C) Evaluation of ROS levels (*η* ^2^ = 0.835, 95% CI 55.185–73.407) by kits. (D) Evaluation of pyroptosis (PI^+^Caspase 1^+^) (*η* ^2^ = 0.842, 95% CI 15.858–20.581) by flow cytometry. Cell experiments were independently replicated thrice. Data were expressed as mean ± SD. One‐way ANOVA was utilized to compare data among multiple groups, followed by Tukey's multiple comparison test. *p* < 0.05 was considered statistically significant. D, DMSO; N, Nigericin; TZ, terazosin.

To further investigate the role of the NLRP3 inflammasome, HT‐22 cells were treated with TZ in combination with the NLRP3 activator Nigericin, followed by OGD/R stimulation. Compared with the OGD/*R* + TZ + DMSO2 group, the OGD/*R* + TZ + *N* group exhibited significantly increased expression of NLRP3, ASC, and cleaved caspase‐1 proteins. In addition, the levels of IL‐1β and IL‐18 in the cell supernatant were markedly elevated, accompanied by enhanced ROS production and elevated GSDMD‐N protein expression, indicating aggravated pyroptosis (Figure 6A–E).

Collectively, these results suggest that activation of the NLRP3 inflammasome partially attenuates the protective effect of TZ against OGD/R‐induced pyroptosis in HT‐22 cells.

### 3.7 TZ Suppressed Neuronal Pyroptosis in the Hippocampal CA1 Region of CI/RI Mice Through Inhibition of the ROS/NLRP3 Pathway

**FIGURE 7:**
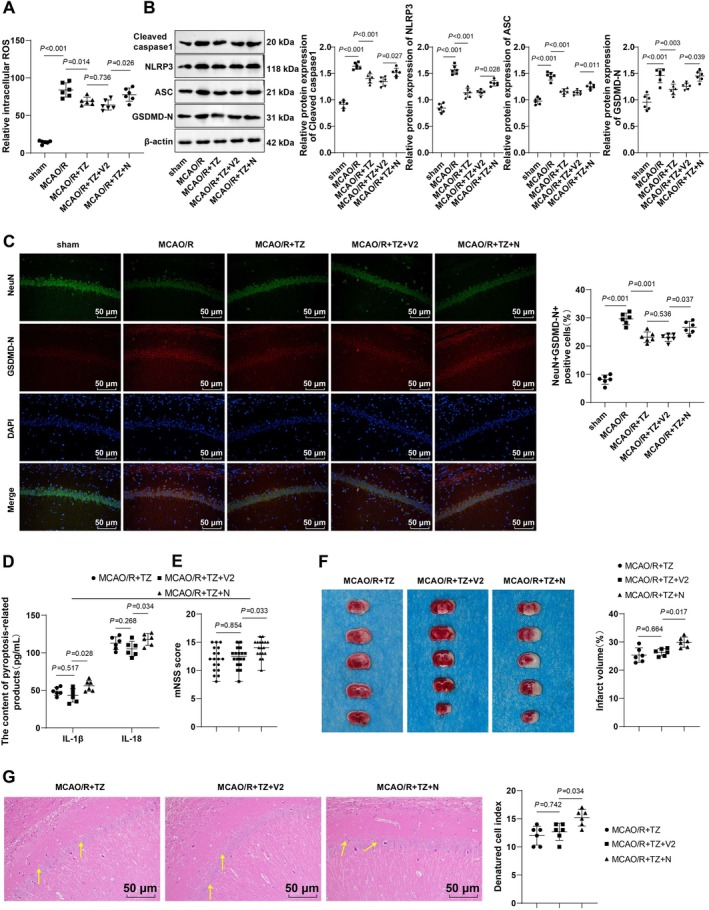
TZ improved CI/RI by curbing the ROS/NLRP3 pathway to decrease neuronal pyroptosis. (A) Evaluation of ROS levels (*η* ^2^ = 0.935, 95% CI 52.059–71.616) by kits, *n* = 6. (B) Measurement of NLRP3 (*η* ^2^ = 0.932, 95% CI 1.106–1.292), ASC (*η* ^2^ = 0.911, 95% CI 1.139–1.266), Cleaved caspase‐1 (*η* ^2^ = 0.906, 95% CI 1.272–1.469), and GSDMD‐N (*η* ^2^ = 0.662, 95% CI 1.209–1.389) protein levels by western blot, *n* = 6. (C) Immunofluorescence staining for neuronal pyroptosis (NeuN+GSDMD‐N+) (*η* ^2^ = 0.952, 95% CI 19.197–24.979), GSDMD‐N (green), NeuN (red), and DAPI (blue), *n* = 6. (D) Measurement of IL‐1β (*η* ^2^ = 0.378, 95% CI 44.975–53.664) and IL‐18 (*η* ^2^ = 0.286, 95% CI 107.074–116.807) levels by ELISA, *n* = 6. (E) The mNSS score used for evaluating neurological deficits (*η* ^2^ = 0.157, 95% CI 12.009–13.139), *n* = 18. (F) Representative TTC‐stained coronal brain sections and quantitative analysis of the cerebral infarction volume (*η* ^2^ = 0.490, 95% CI 25.553–28.311), *n* = 6. (G) H&E staining showing pathological tissue damage in the hippocampal CA1 region (*η* ^2^ = 0.480, 95% CI 12.271–14.284), *n* = 6. One‐way ANOVA was used for data comparisons between multiple groups, and Tukey's multiple comparison test was used for the post hoc test. *p* < 0.05 was considered statistically significant. N, Nigericin; TZ, terazosin; V, Vehicle.

To validate these findings in vivo, mice were intraperitoneally injected with TZ and 4 mg/kg Nigericin prior to MCAO/R modeling. Compared with the sham group, MCAO/R mice exhibited elevated ROS levels and increased expression of NLRP3, ASC, and cleaved caspase‐1 proteins in the hippocampal CA1 region, indicating activation of the ROS/NLRP3 signaling pathway. Notably, TZ treatment significantly suppressed ROS accumulation and reduced the expression of NLRP3‐related proteins compared with the MCAO/R group (Figure 7A,B).

Furthermore, compared with the MCAO/*R* + TZ + V2 group, the MCAO/*R* + TZ + *N* group showed partial activation of the ROS/NLRP3 pathway in the hippocampal CA1 region. This was accompanied by increased neuronal pyroptosis (NeuN^+^GSDMD‐N^+^), elevated levels of inflammatory cytokines (IL‐18 and IL‐1β), and higher expression of the pyroptosis marker GSDMD‐N (Figure 7A–D). In addition, activation of NLRP3 partially reversed the protective effects of TZ on CI/RI in mice (Figure 7E–G).

Taken together, these findings indicate that TZ alleviates CI/RI by inhibiting the ROS/NLRP3 signaling pathway and reducing neuronal pyroptosis in the hippocampal CA1 region.

## 4 Discussion

In the present study, we demonstrated that TZ alleviated CI/RI in a dose‐dependent manner in the MCAO/R mouse model and attenuated OGD/R‐induced neuronal pyroptosis in HT‐22 cells by enhancing mitophagy. Mechanistically, TZ exerted neuroprotective effects, at least in part, through a mitophagy‐mediated reduction in ROS accumulation, suppression of NLRP3 inflammasome activation, and inhibition of pyroptosis.

TZ is a classical α1‐AR antagonist that has been reported to reduce organ injury and improve survival in rodent models of stroke. In addition, TZ exhibits protective effects in several degenerative disorders and is of particular interest for neurological diseases because it can cross the blood–brain barrier [12]. In our study, TZ showed the strongest neuroprotective effect at 10 μg/kg, whereas the effect was weakened at 100 μg/kg. This biphasic response is consistent with previous observations that higher doses of TZ reduce ATP levels and thereby compromise neuroprotection [12]. Because ATP preservation is critical for resistance to ischemic injury [22], the diminished efficacy observed at 100 μg/kg may reflect energy depletion and subsequent disruption of neuronal homeostasis. On this basis, 10 μg/kg was selected as the optimal dose for subsequent experiments.

Given that TZ competitively blocks α1‐AR activation and prevents norepinephrine‐induced increases in intracellular Ca^2+^ levels, and that excessive intracellular Ca^2+^ concentration promotes mitochondrial dysfunction and neuronal injury [23], we examined whether α1‐AR antagonism contributed to its protective effects. Rescue experiments with the α1‐adrenergic receptor agonist phenylephrine showed no significant differences between the MCAO/*R* + TZ group and the group treated with both TZ and PE, supporting the conclusion that TZ‐mediated neuroprotection is closely associated with α1‐AR antagonism.

A growing body of evidence indicates that TZ preserves mitochondrial integrity, improves mitochondrial function, and restores impaired autophagic flux. For example, TZ promotes mitophagy in mice with non‐alcoholic fatty pancreas disease [14], and mechanistic studies have shown that it enhances mitophagy through GPR119 activation and inhibition of the MST1‐FOXO3a pathway [14]. Consistent with these findings, our in vivo and in vitro experiments demonstrated that TZ increased mitophagy, as demonstrated by enhanced MitoTracker‐LC3B colocalization, increased LC3B II/I and Beclin‐1 expression, and decreased p62 levels. TZ also restored mitochondrial membrane potential and reduced intracellular ROS production. Importantly, these neuroprotective effects were markedly blunted by the mitophagy inhibitor Mdivi‐1, indicating that mitophagy is a central mediator of TZ‐induced neuroprotection. Together, these findings suggest that TZ promotes the clearance of dysfunctional mitochondria, limits ROS accumulation, and prevents downstream activation of the NLRP3 inflammasome. This interpretation is further supported by studies showing that microglial‐specific MST1 deficiency attenuates ischemic injury [24] and that induction of mitophagy by β‐asarone protects against CI/RI through the PINK1/Parkin pathway [25].

Pyroptosis was evaluated in the present study using NeuN/GSDMD‐N double immunofluorescence. TZ enhanced mitophagy in the hippocampal CA1 region and reduced neuronal pyroptosis, as reflected by decreased NeuN^+^GSDMD‐*N* ^+^ cells, lower GSDMD‐N expression, and reduced IL‐18 and IL‐1β levels. Similar anti‐pyroptotic effects of TZ have been reported in ulcerative colitis models, where TZ suppresses IL‐1β and IL‐18 release, and reduces caspase‐1 and GSDMD expression [13]. In our CI/RI model, co‐administration of TZ and the mitophagy inhibitor Mdivi‐1 significantly increased neuronal pyroptosis and partially abolished the protective effects of TZ in vivo. Our in vitro data further showed that TZ restored mitochondrial membrane potential, enhanced mitophagy, and inhibited OGD/R‐induced pyroptosis, whereas inhibition of mitophagy reversed these effects. These results strongly support the conclusion that TZ suppresses neuronal pyroptosis through a mitophagy‐dependent mechanism.

Because ROS reduction has been identified as an important mechanism of TZ‐related neuroprotection in Parkinson's disease [26], we further explored whether TZ regulates the ROS/NLRP3 axis in CI/RI. Our data showed that TZ inhibited OGD/R‐induced NLRP3 inflammasome activation in HT‐22 cells by enhancing mitophagy and limiting ROS accumulation. Similar mechanisms have been observed with other compounds, such as apigenin, which induces mitophagy and suppresses ROS‐dependent NLRP3 inflammasome activation [27]. Since NLRP3 activation drives IL‐1β secretion and pyroptosis and contributes to neuroinflammation [28], its inhibition likely represents a major component of TZ‐mediated neuroprotection. Consistent with this interpretation, activation of the NLRP3 inflammasome partially reversed the inhibitory effect of TZ. Notably, inhibition of mitophagy with Mdivi‐1 or activation of the NLRP3 inflammasome with Nigericin only partially reduced the protective effects of TZ, suggesting that other mechanisms are also involved. Previous studies have reported that TZ can activate the Nrf2 or ERK1/2 signaling pathways and thereby suppress oxidative stress [29], and that it can bind phosphoglycerate kinase 1 (Pgk1) to promote glycolysis and neuronal protection in PD models [26, 30]. These findings suggest that TZ may exert neuroprotective effects through additional mechanisms that extend beyond α1‐AR antagonism.

Importantly, inhibition of the ROS/NLRP3/pyroptosis axis is increasingly recognized as a promising therapeutic strategy for CI/RI [31]. Supporting this concept, icariside II alleviates neuronal degeneration and neuroinflammation after CI/RI by suppressing ROS/NLRP3‐mediated pyroptosis [31]. To our knowledge, the present study is the first to demonstrate that TZ protects against CI/RI through modulation of the ROS/NLRP3 signaling pathway. These findings indicate that TZ may represent a promising therapeutic agent for CI/RI by suppressing ROS/NLRP3‐mediated neuronal pyroptosis. Specifically, TZ reduced mitochondrial ROS production, preserved mitochondrial function, and delayed activation of the NLRP3 inflammasome, collectively supporting its potential as a neuroprotective agent.

Several limitations should be acknowledged. First, the precise molecular mechanism through which TZ regulates mitophagy remains unclear, and the possibility that it modulates additional inflammasome pathways or signaling pathways requires further investigation. Second, our study focused on the acute phase of injury at 24 h post‐reperfusion, a time point at which infarct burden, neurological deficits, cerebral infarction, and pathological damage typically peak during this period [32]. This period is also associated with robust oxidative stress, inflammation, autophagic activation, and neuronal apoptosis [33, 34]. Clinical evidence further indicates that neuroinflammatory changes within the first 24 h after thrombolysis are strongly associated with infarct expansion and long‐term functional impairment [35]. Earlier time points (6–12 h) mainly reflect the initial amplification of injury, whereas later stages (3–7 days) involve compensatory repair processes, including metabolic recovery and neuroregeneration [36]. Therefore, 24 h after reperfusion was selected to ensure reliable and reproducible assessment of acute injury. However, the present study did not determine whether the acute neuroprotective effects of TZ persist over the long term, nor did it evaluate its effects on neural repair or potential toxicity in visceral organs. Moreover, translation of these findings into clinical practice will require further investigation of critical parameters, including optimal dosage and therapeutic time windows. Future studies should incorporate RNA sequencing or other multi‐omics approaches to comprehensively characterize the TZ‐Ca^2+^‐mitophagy‐pyroptosis regulatory axis and identify the key mitophagy pathways through which TZ influences neuronal survival after CI/RI injury. Time‐course studies at 3, 7, and 14 days after reperfusion, together with earlier time points at 6 and 12 h hours, will further clarify the onset and durability of TZ‐mediated neuroprotection. Finally, because intraperitoneal injection has limitations in terms of delivery efficiency and tissue targeting. Systemic drug distribution may reduce the effective concentration reaching the brain and potentially compromise intervention during the optimal therapeutic window for CI/RI [37]. Therefore, in subsequent experiments, intracranial stereotaxic injection will be employed to administer the NLRP3 agonist nigericin into the hippocampal CA1 region, allowing more accurate assessment of its effects on NLRP3 inflammasome and neuronal pyroptosis.

## Ethics Statement

All animal experiments were conducted in accordance with the Guide for the Care and Use of Laboratory Animals (NIH Publication No. 8023, revised in 1978) and were approved by the Institutional Animal Care and Use Committee of The First Affiliated Hospital of Jinan University (Ethical Approval No.: 2024‐053, Approval date: March 10, 2024). All procedures strictly adhered to the approved protocols, with particular attention given to minimizing animal numbers and alleviating animal suffering.

## Conflicts of Interest

The authors declare no conflicts of interest.

## Supporting information


**Figure S1:** Post hoc power analysis was conducted using General Power Analysis Software.

**Data S1:** kjm270242‐sup‐0002‐WB.pdf.

## Data Availability

The data that support the findings of this study are available from the corresponding author upon reasonable request.
