# Supplementary material for: Terazosin Attenuates Neuronal Pyroptosis by Regulating the Mitochondrial ROS/NLRP3 Inflammasome Axis Through Mitophagy in Cerebral Ischemia–Reperfusion Injury
Source: Kaohsiung J Med Sci. 2026 Jun 9:e70242. Online ahead of print. doi: 10.1002/kjm2.70242 (PMC13399670; doi:10.1002/kjm2.70242)
Supplement: Supplementary file 1 — Figure S1: Post hoc power analysis was conducted using General Power Analysis Software. [file KJM2-9999-e70242-s001.docx]

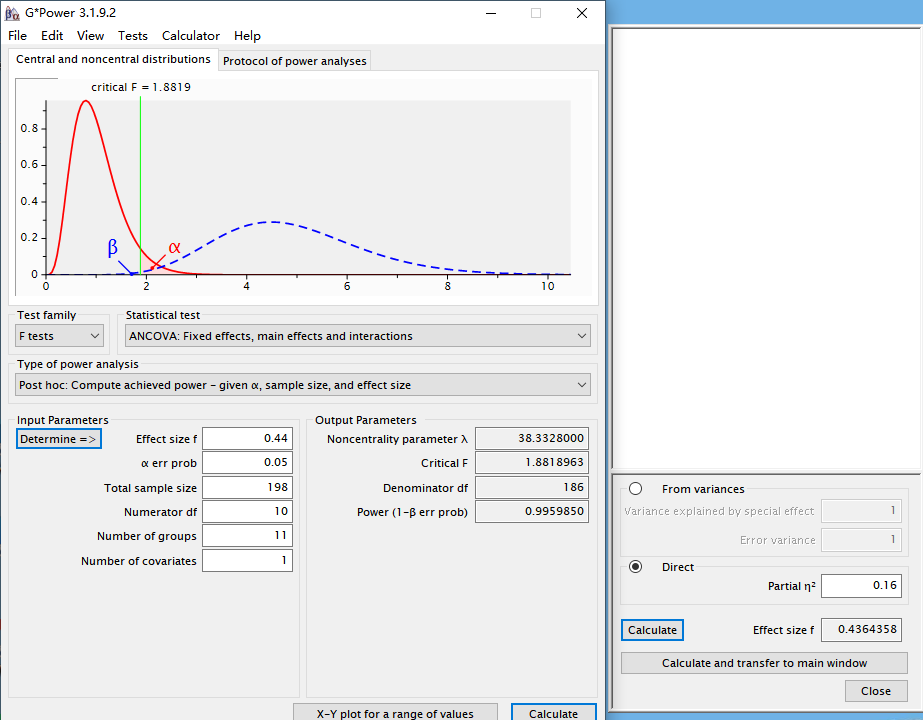


**Supplementary Figure 1** Post hoc power analysis was conducted using General Power Analysis Software.
