# Supplementary material for: Terazosin Attenuates Neuronal Pyroptosis by Regulating the Mitochondrial ROS/NLRP3 Inflammasome Axis Through Mitophagy in Cerebral Ischemia–Reperfusion Injury
Source: Kaohsiung J Med Sci. 2026 Jun 9:e70242. Online ahead of print. doi: 10.1002/kjm2.70242 (PMC13399670; doi:10.1002/kjm2.70242)
Supplement: Supplementary file 2 — Data S1: kjm270242‐sup‐0002‐WB.pdf. [file KJM2-9999-e70242-s002.pdf]

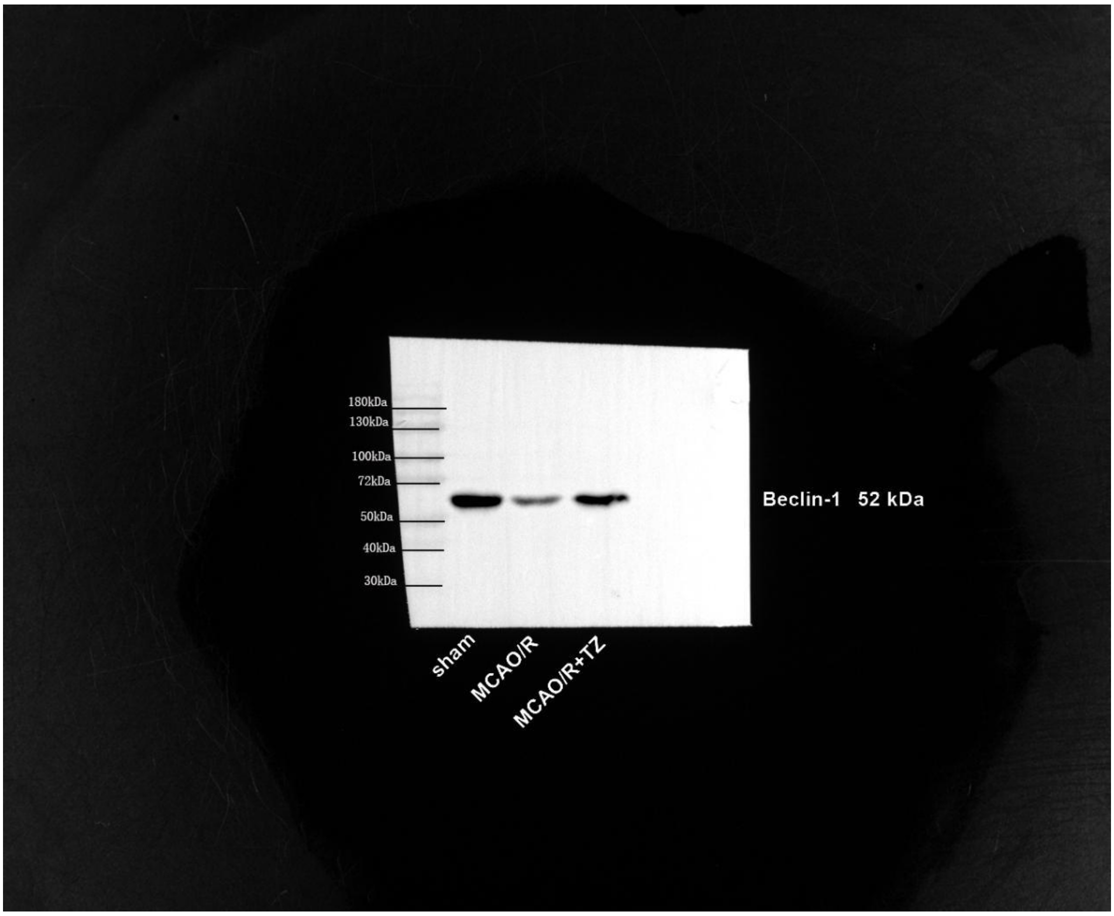

180kDa
130kDa
100kDa
72kDa
50kDa
40kDa
30kDa
Beclin-1 52 kDa
sham
MCAO/R
MCAO/R+TZ

2D-Beclin-1

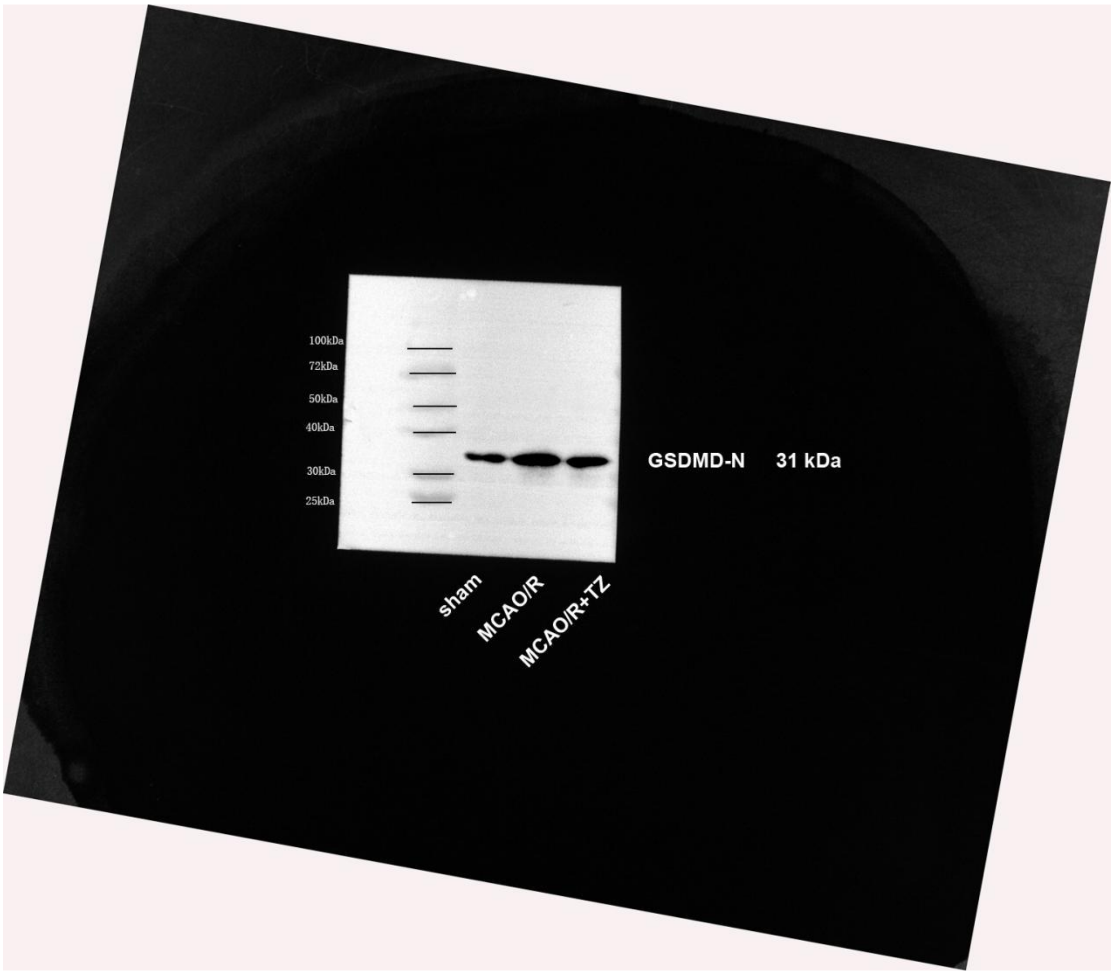
100kDa
72kDa
50kDa
40kDa
30kDa
25kDa
GSDMD-N 31 kDa
sham
MCAO/R
MCAO/R+TZ

2D-GSDMD-N

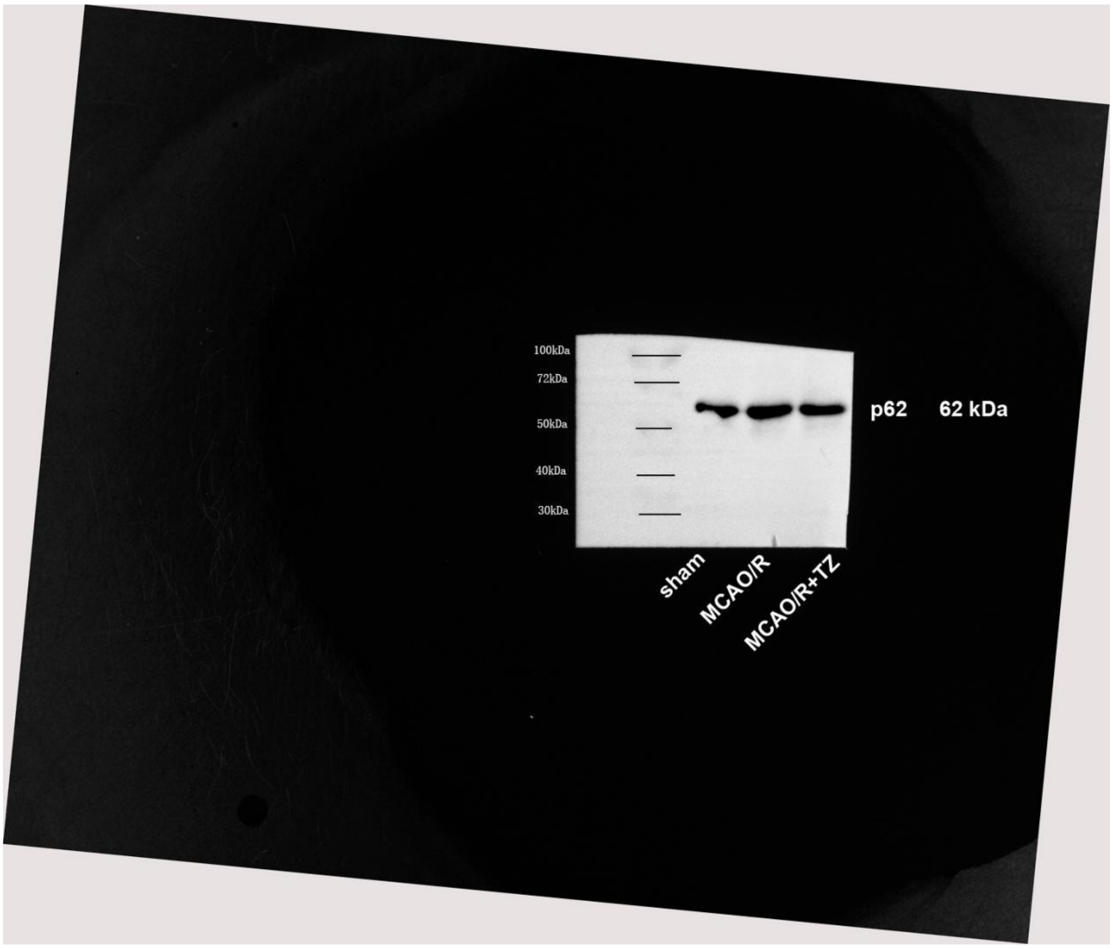

100kDa
72kDa
50kDa
40kDa
30kDa
p62
62 kDa
sham
MCAO/R
MCAO/R+TZ

2D-p62

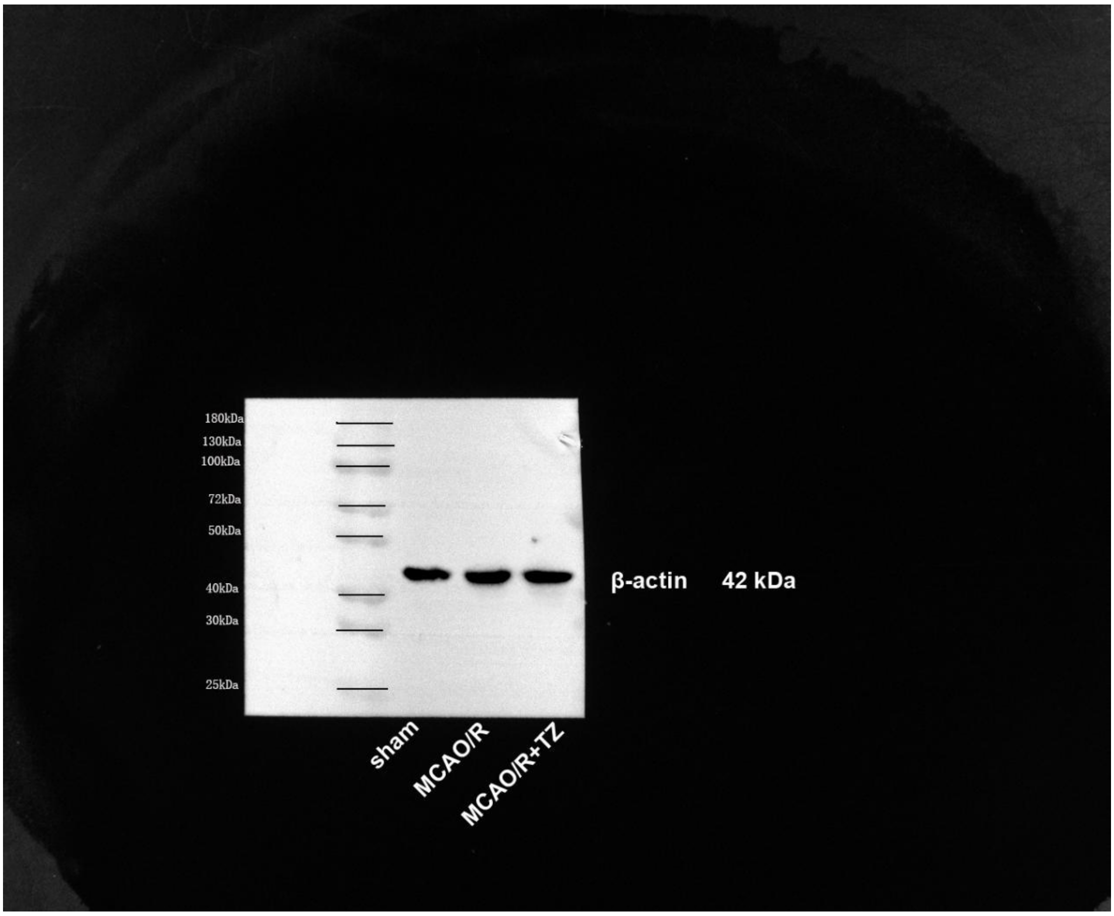
180kDa
130kDa
100kDa
72kDa
50kDa
40kDa
30kDa
25kDa
β-actin 42 kDa
sham
MCAO/R
MCAO/R+TZ

2D-β-actin

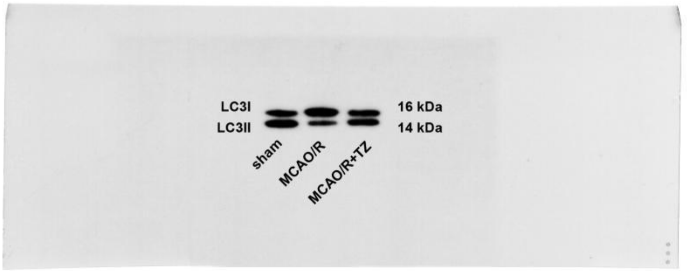

LC3B

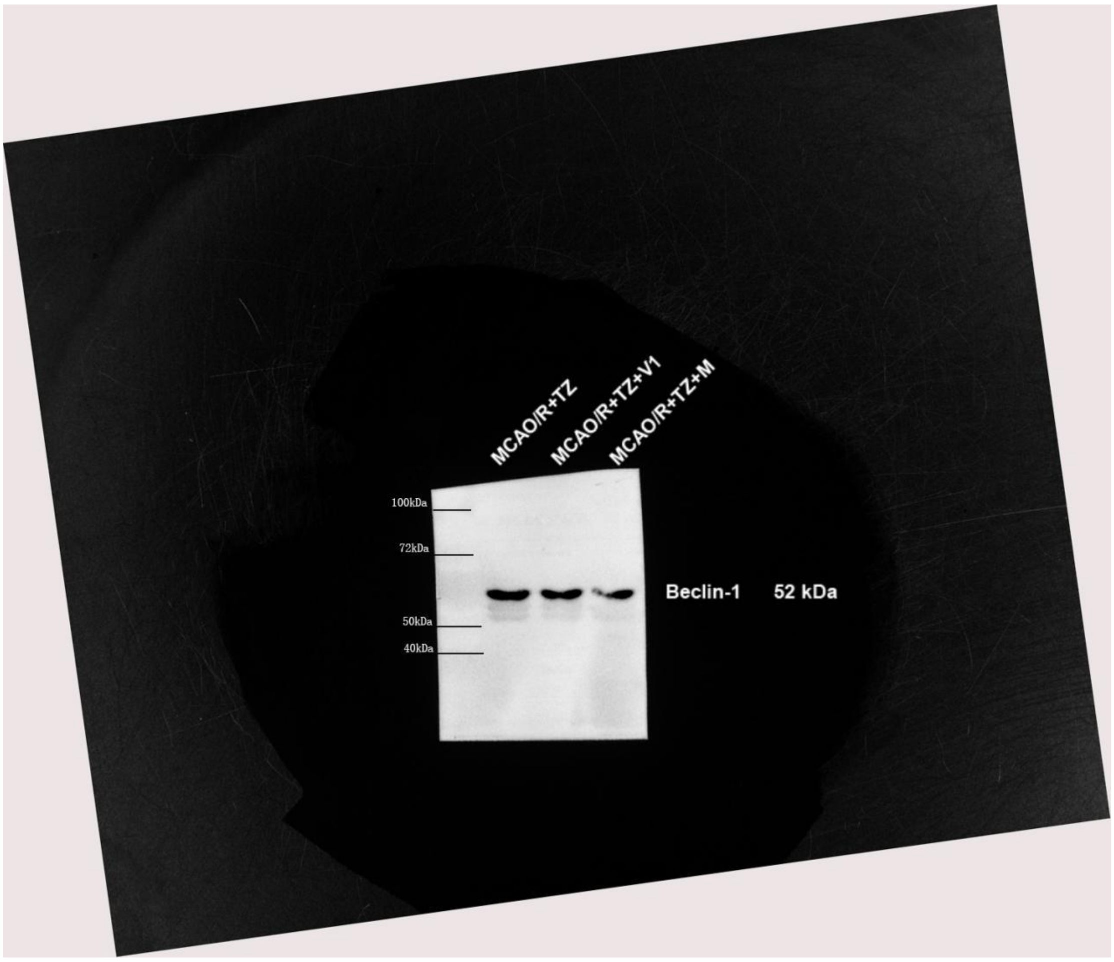
MCAO/R+TZ
MCAO/R+TZ+V1
MCAO/R+TZ+M
100kDa
72kDa
50kDa
40kDa
Beclin-1
52 kDa

3D-Beclin-1

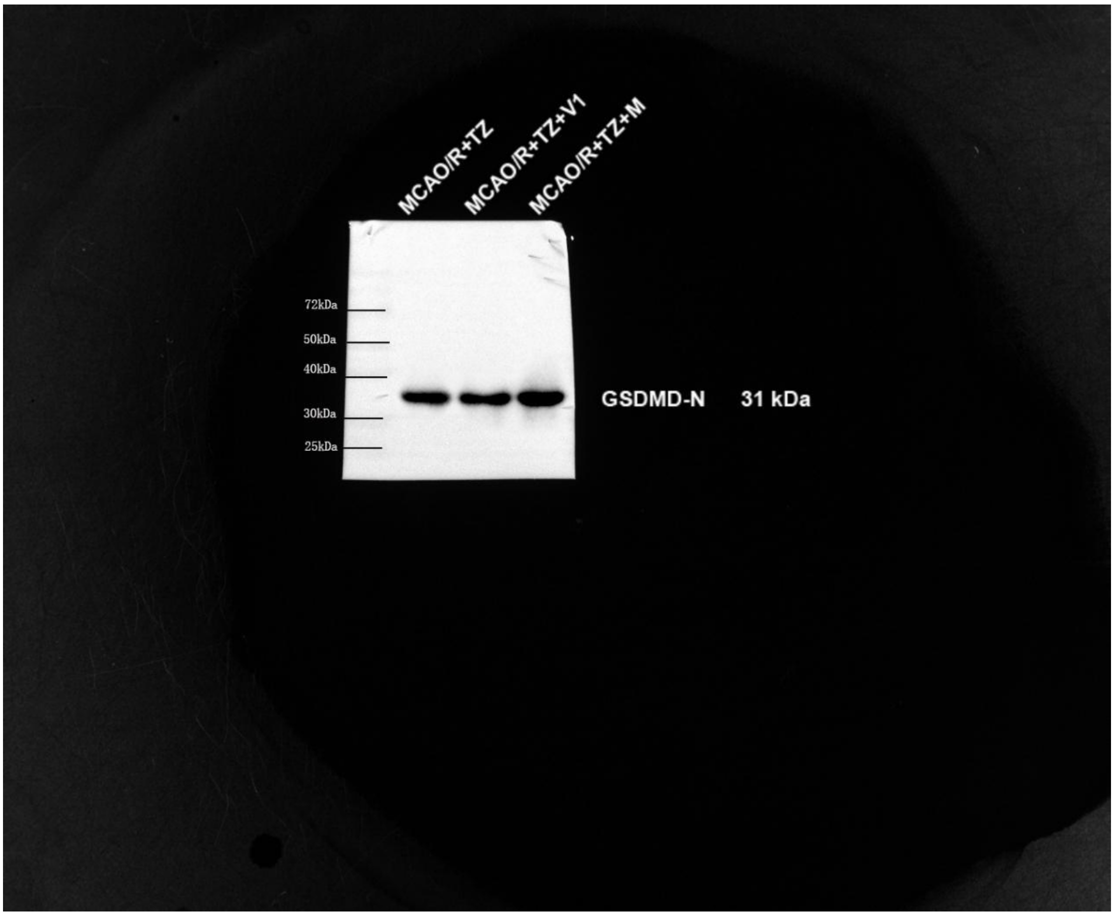
MCAO/R+TZ
MCAO/R+TZ+V1
MCAO/R+TZ+M
72kDa
50kDa
40kDa
30kDa
25kDa
GSDMD-N 31 kDa

3D-GSDMD-N

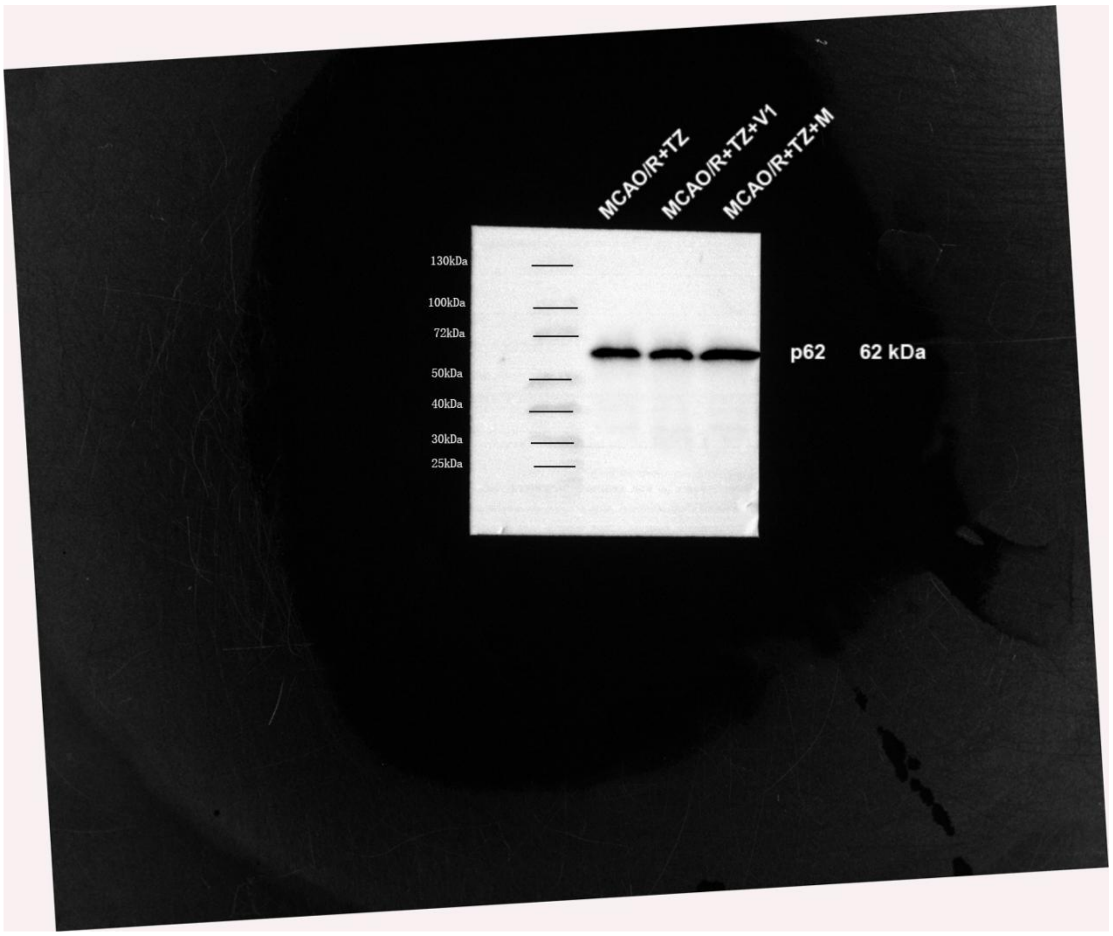
MCAO/R+TZ
MCAO/R+TZ+V1
MCAO/R+TZ+M
130kDa
100kDa
72kDa
50kDa
40kDa
30kDa
25kDa
p62
62 kDa

3D-p62

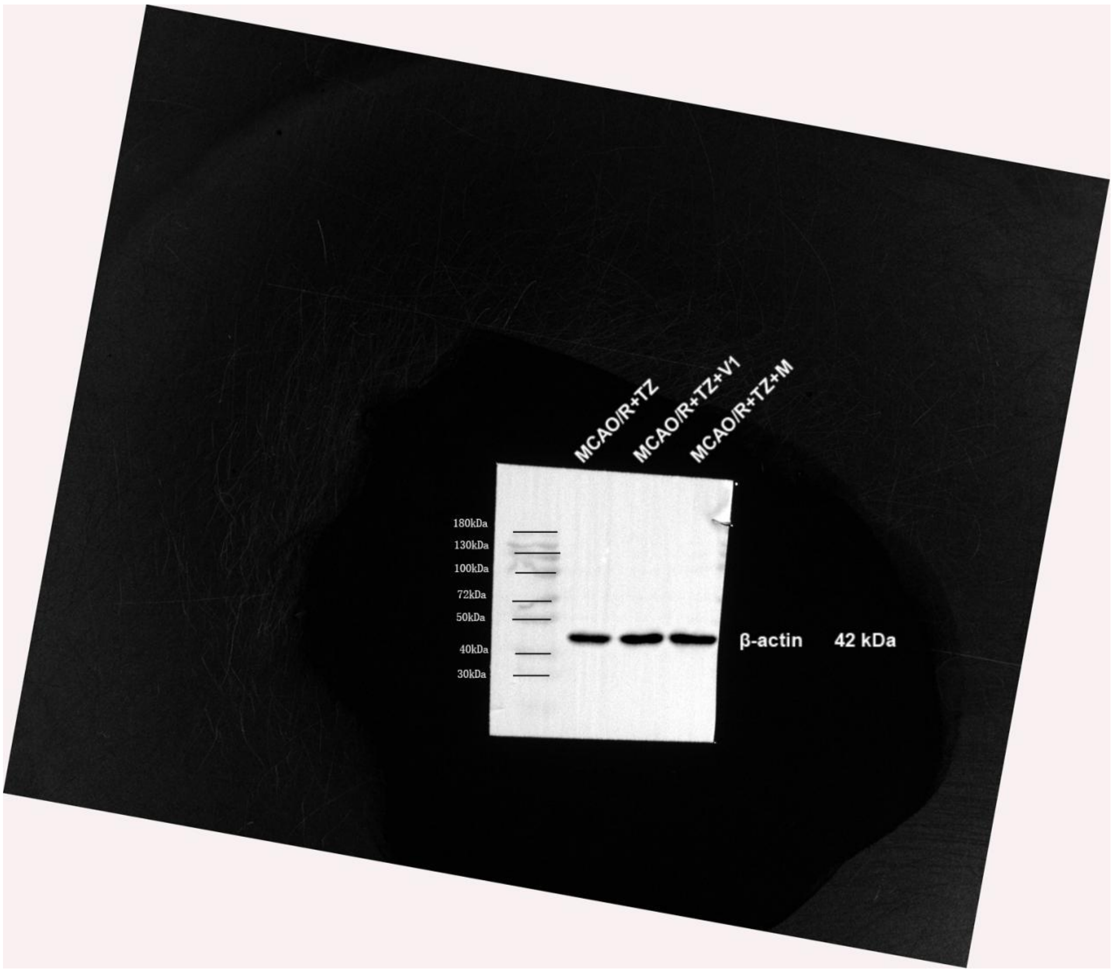

MCAO/R+TZ
MCAO/R+TZ+V1
MCAO/R+TZ+M
180kDa
130kDa
100kDa
72kDa
50kDa
40kDa
30kDa
β-actin
42 kDa

3D-β-actin

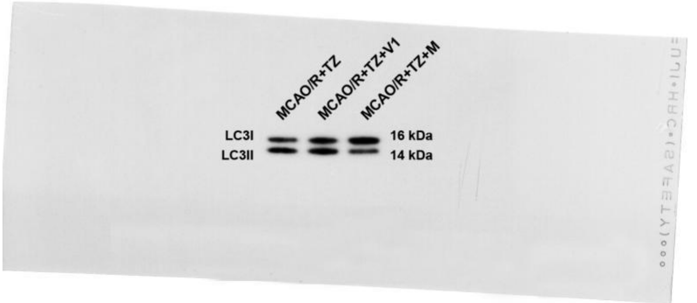

LC3B

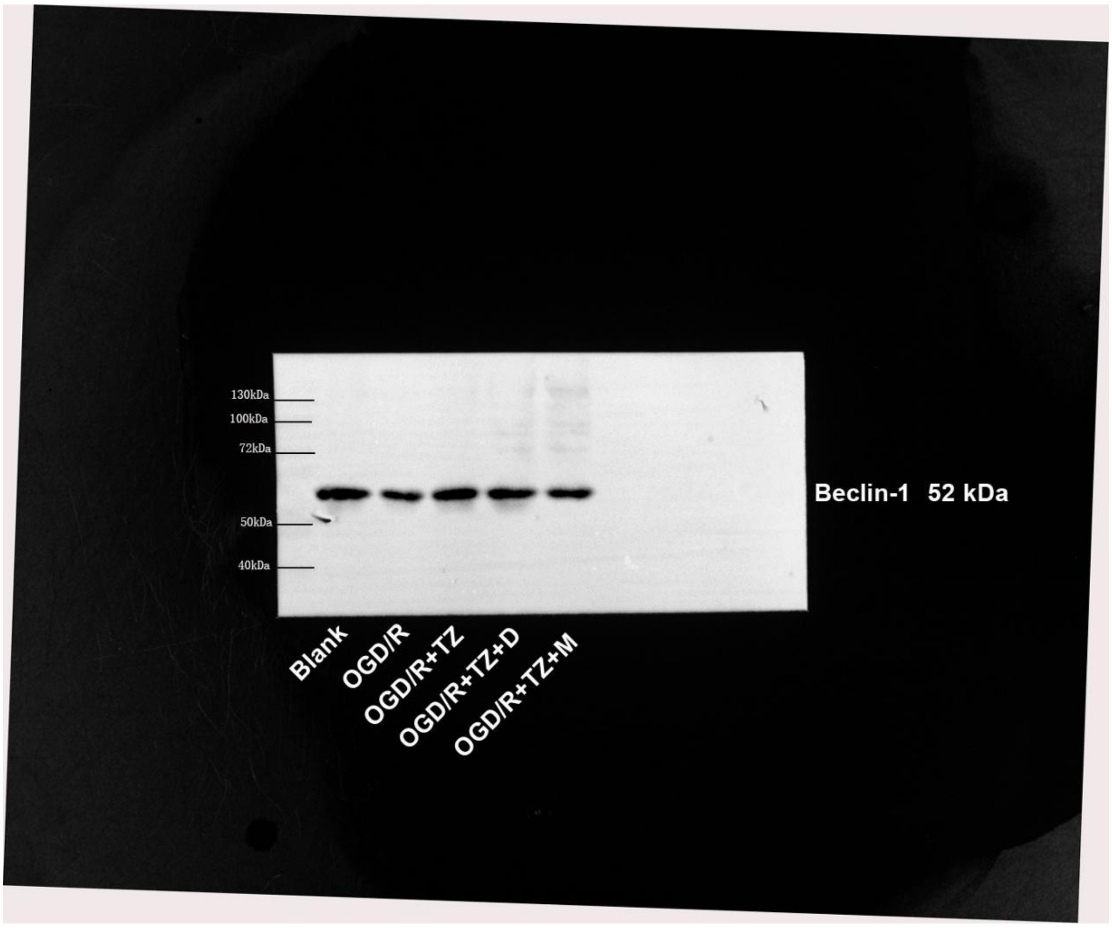
130kDa
100kDa
72kDa
50kDa
40kDa
Beclin-1 52 kDa
Blank
OGD/R
OGD/R+TZ
OGD/R+TZ+D
OGD/R+TZ+M

4F-Beclin-1

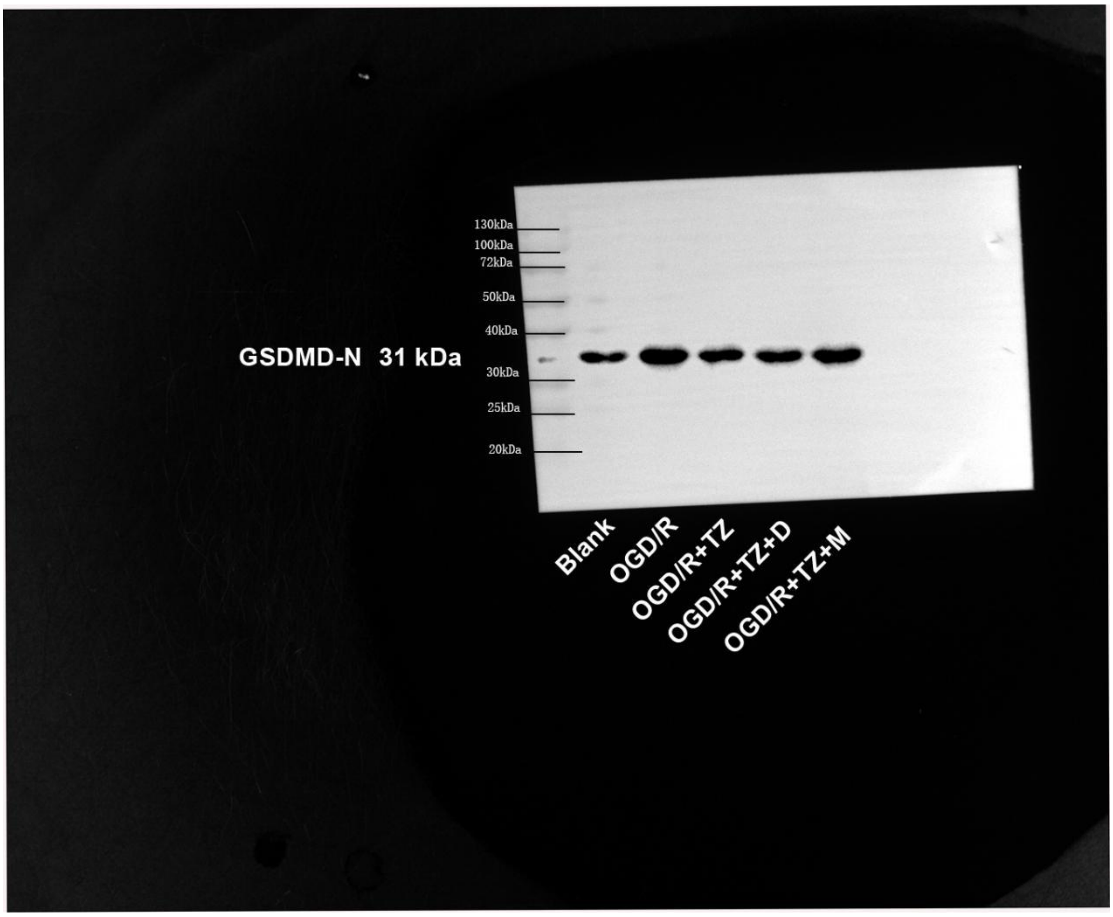
GSDMD-N 31 kDa
130kDa
100kDa
72kDa
50kDa
40kDa
30kDa
25kDa
20kDa
Blank
OGD/R
OGD/R+TZ
OGD/R+TZ+D
OGD/R+TZ+M

4F-GSDMD-N

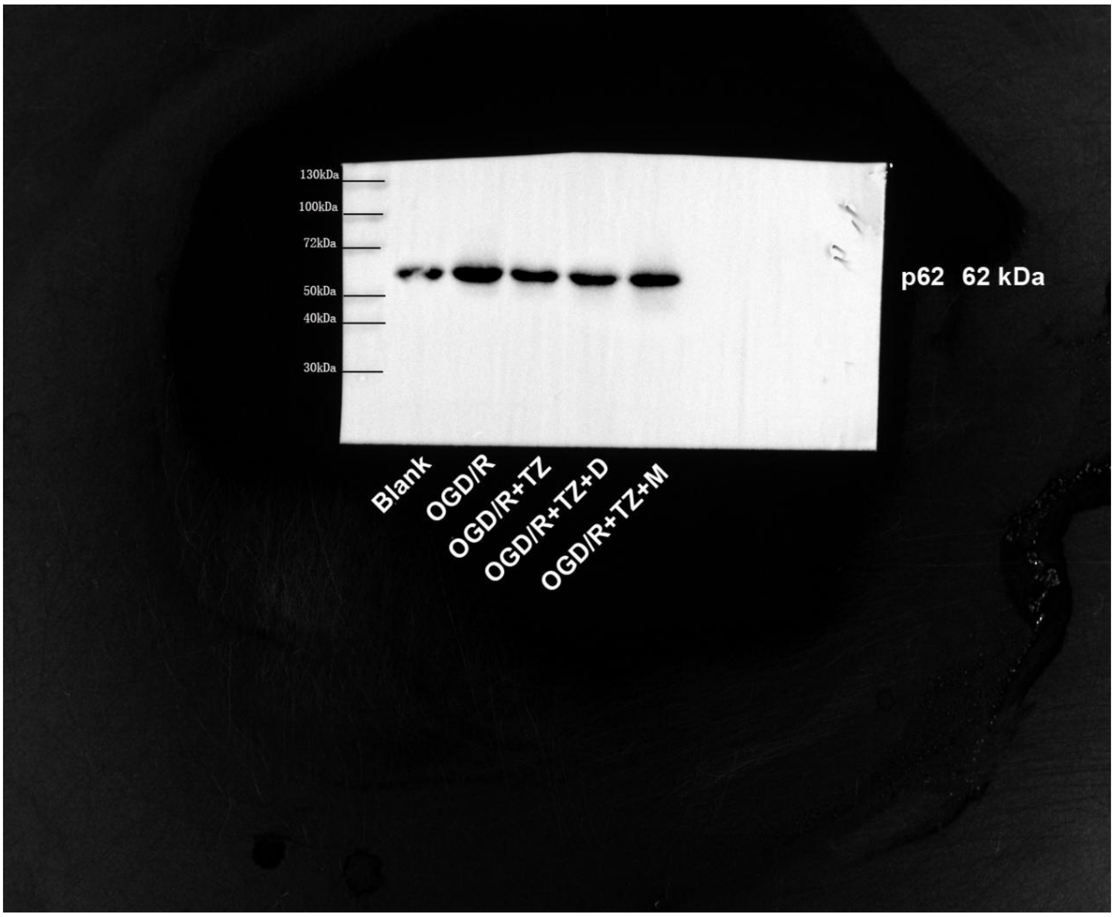

130kDa
100kDa
72kDa
50kDa
40kDa
30kDa
p62 62 kDa
Blank
OGD/R
OGD/R+TZ
OGD/R+TZ+D
OGD/R+TZ+M

4F-p62-011

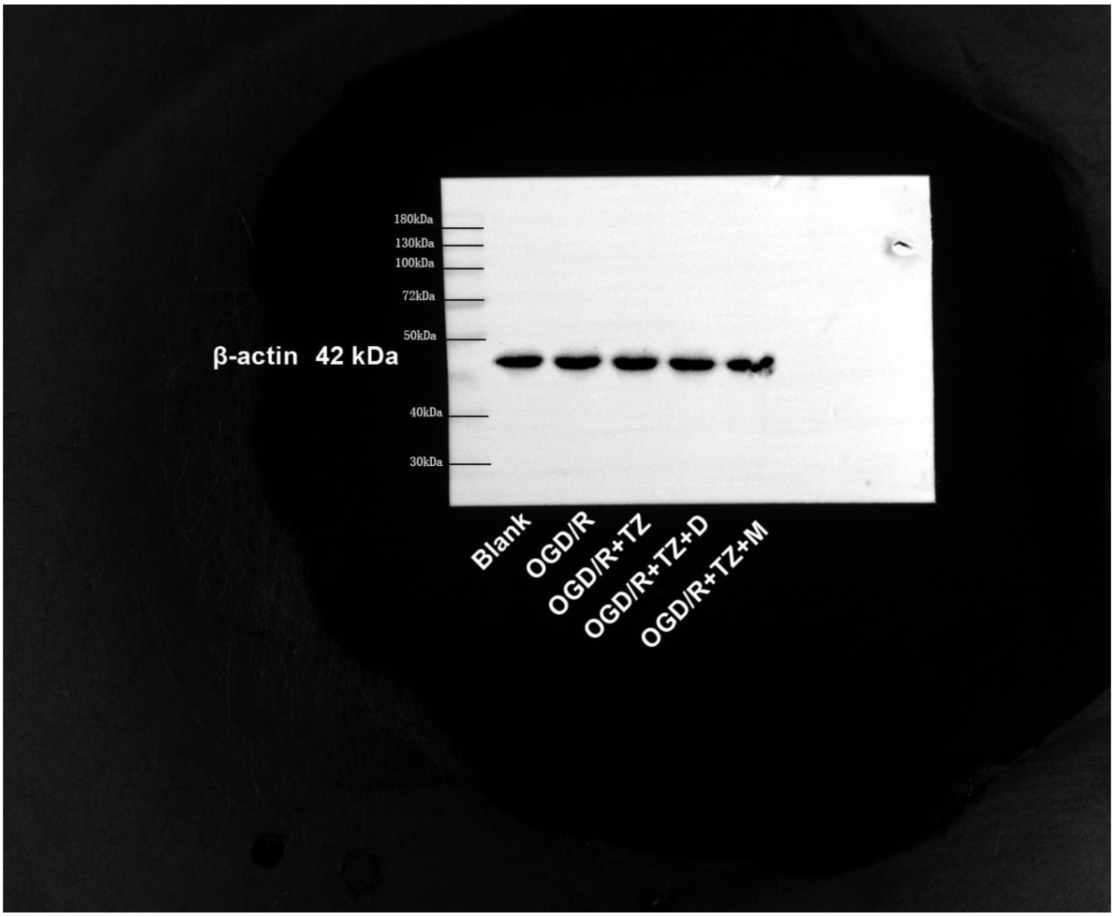
180kDa
130kDa
100kDa
72kDa
50kDa
β-actin 42 kDa
40kDa
30kDa
Blank
OGD/R
OGD/R+TZ
OGD/R+TZ+D
OGD/R+TZ+M

4F-β-actin

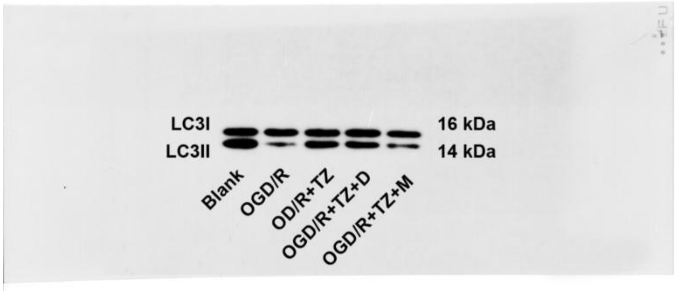

LC3B

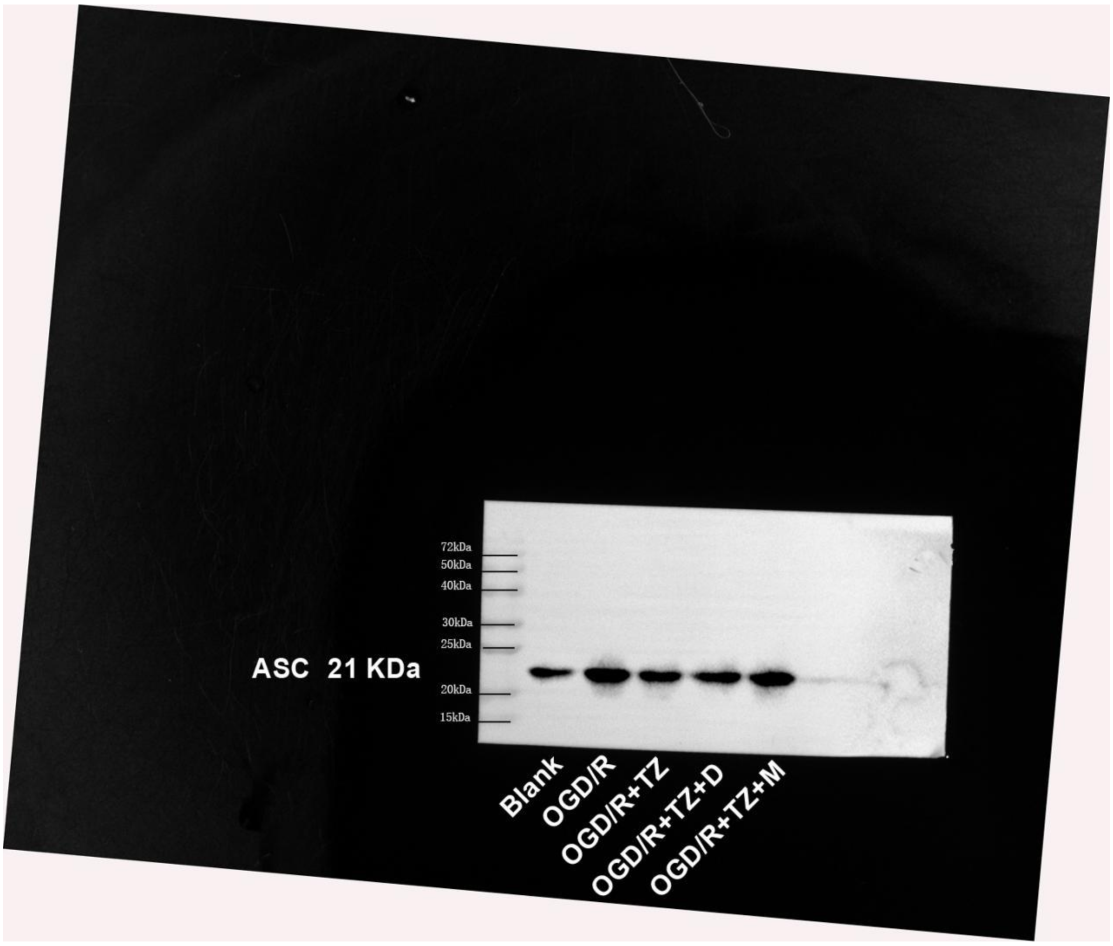

72kDa
50kDa
40kDa
30kDa
25kDa
ASC 21 KDa
20kDa
15kDa
Blank
OGD/R
OGD/R+TZ
OGD/R+TZ+D
OGD/R+TZ+M

5C-ASC

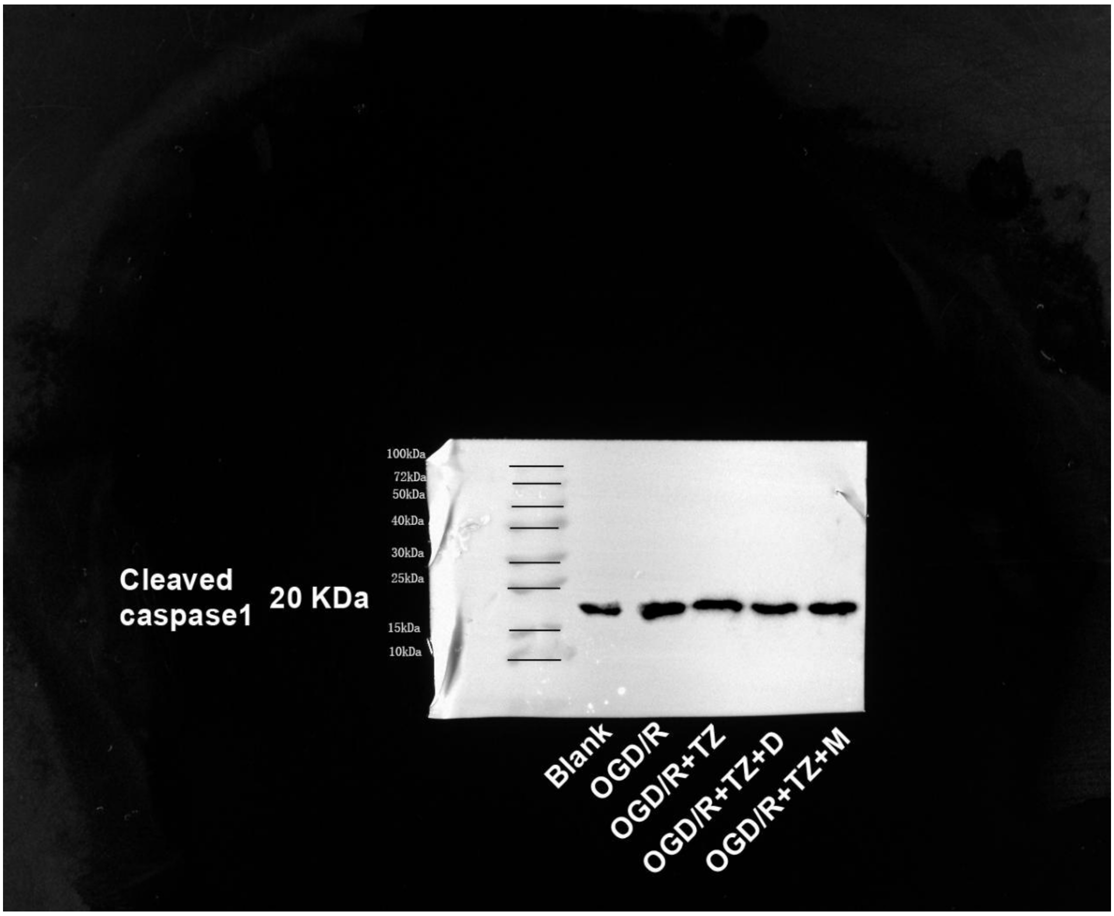

5C-Cleaved caspase1

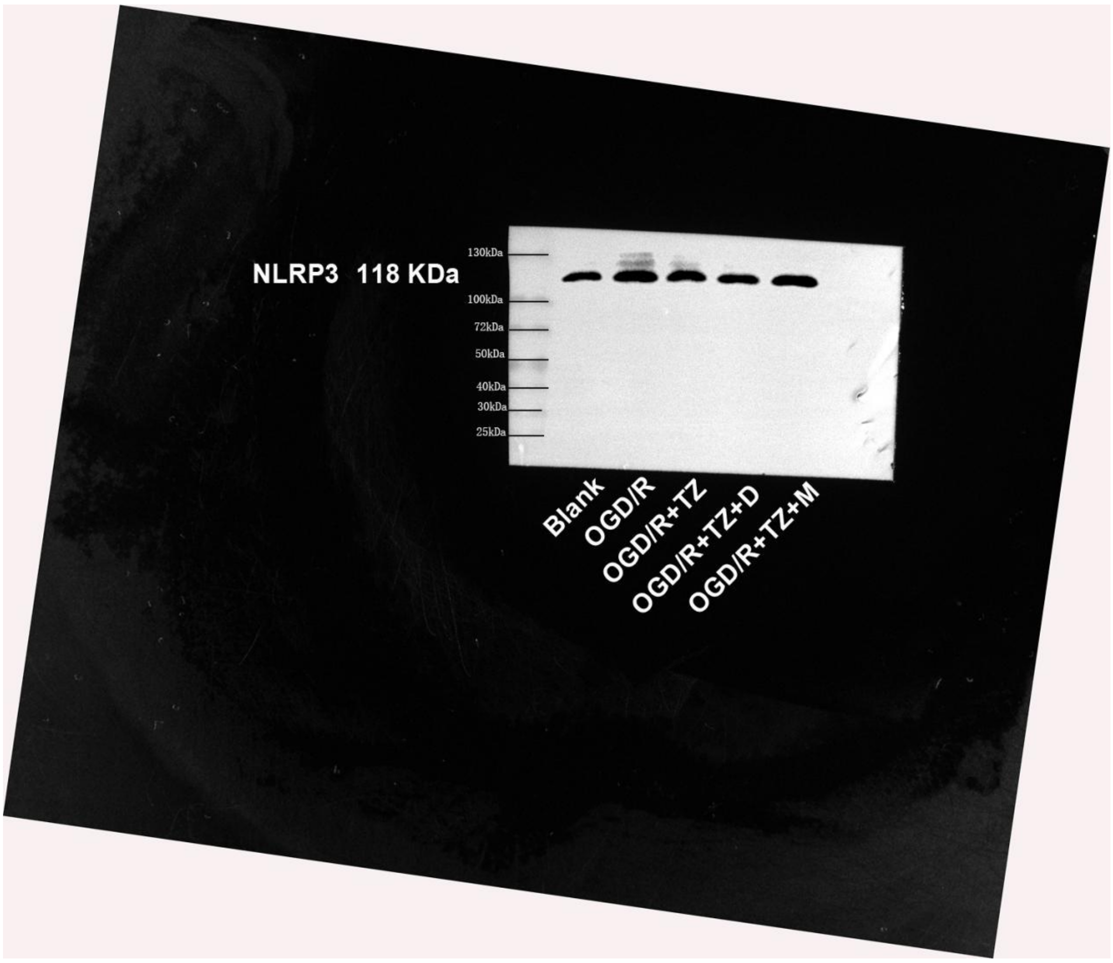
NLRP3 118 KDa
130kDa
100kDa
72kDa
50kDa
40kDa
30kDa
25kDa
Blank
OGD/R
OGD/R+TZ
OGD/R+TZ+D
OGD/R+TZ+M

5C-NLRP3

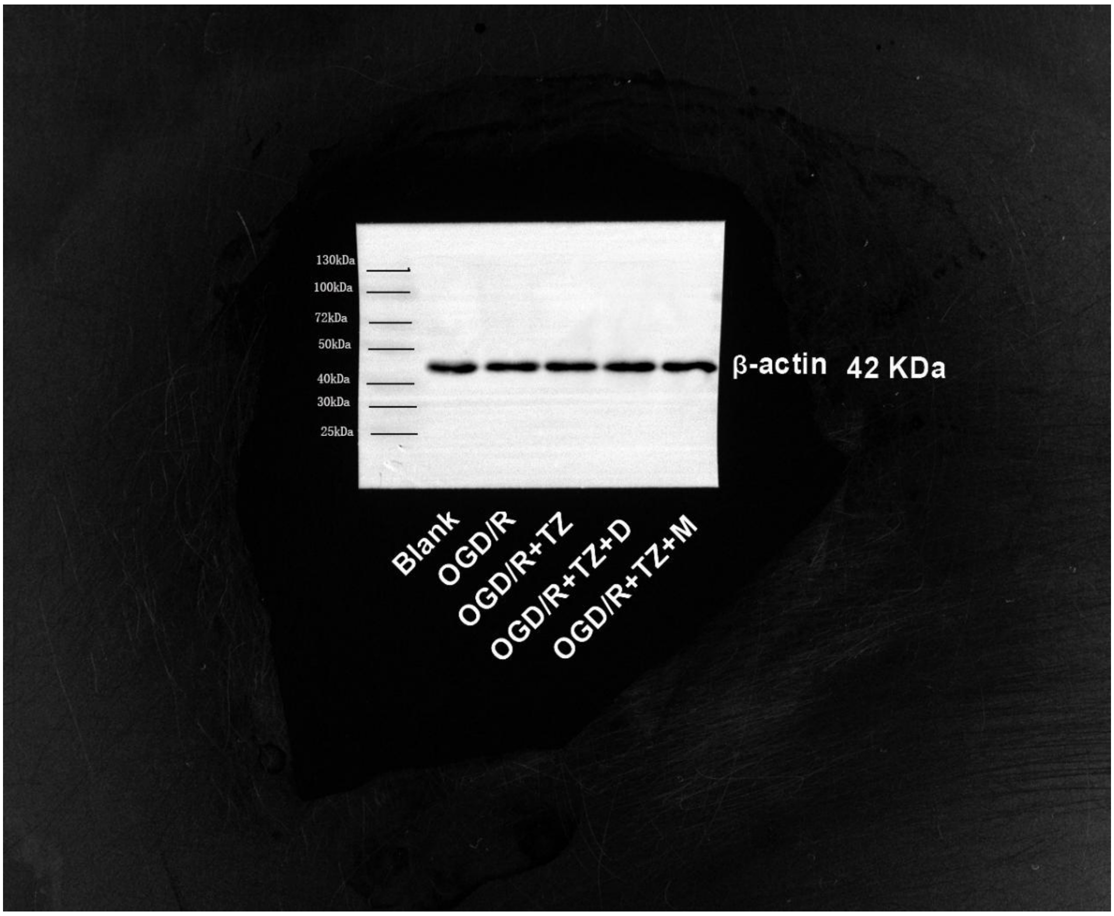
130kDa
100kDa
72kDa
50kDa
40kDa
30kDa
25kDa
β-actin 42 KDa
Blank
OGD/R
OGD/R+TZ
OGD/R+TZ+D
OGD/R+TZ+M

5C-β-actin

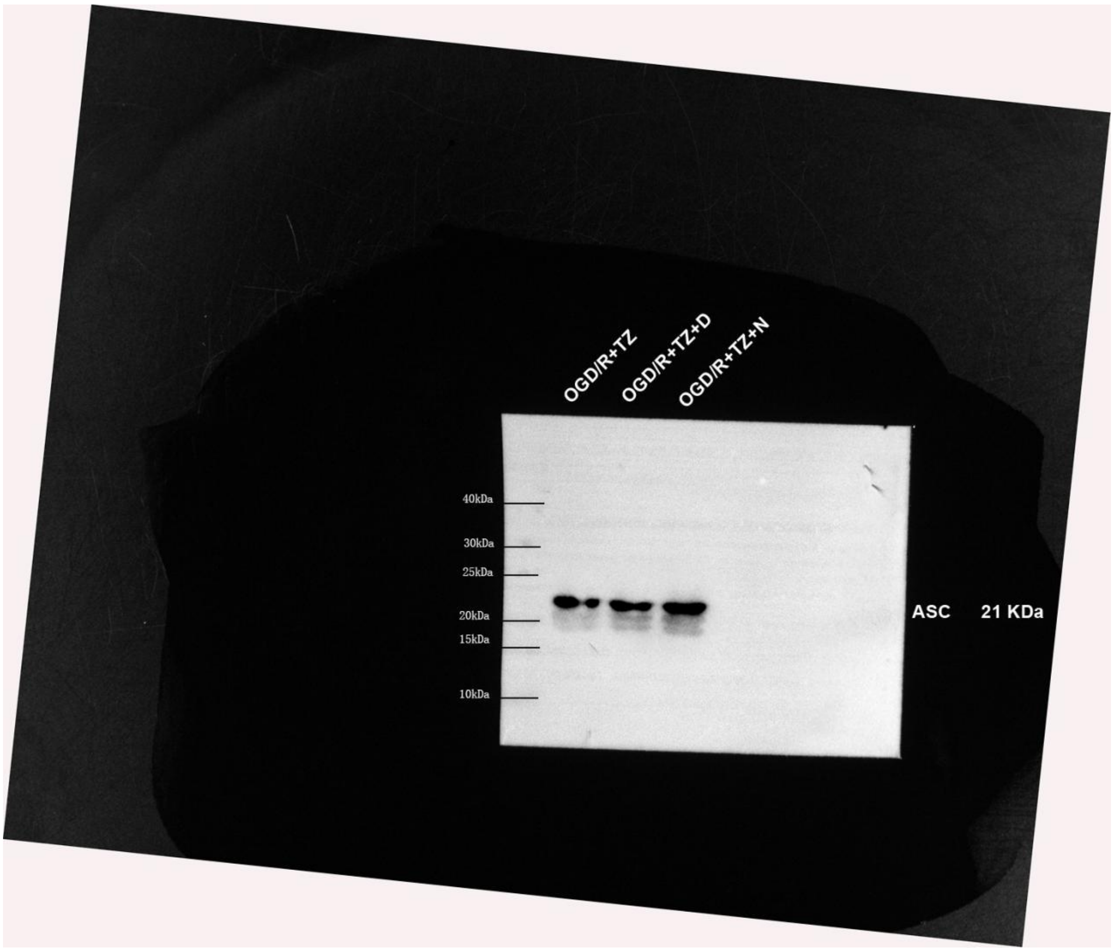

OGD/R+TZ
OGD/R+TZ+D
OGD/R+TZ+N
40kDa
30kDa
25kDa
20kDa
15kDa
10kDa
ASC
21 KDa

6A-ASC

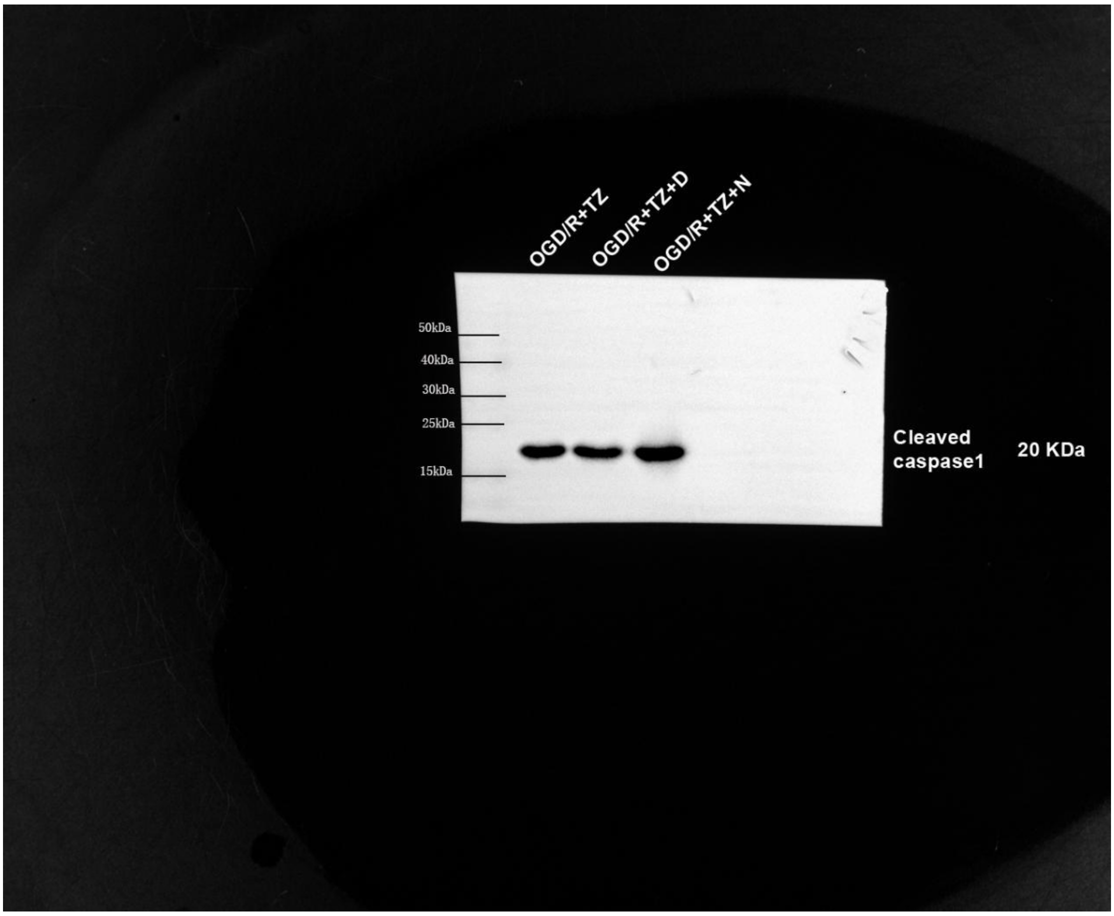
OGD/R+TZ
OGD/R+TZ+D
OGD/R+TZ+N
50kDa
40kDa
30kDa
25kDa
15kDa
Cleaved
caspase1
20 KDa

6A-Cleaved caspase1

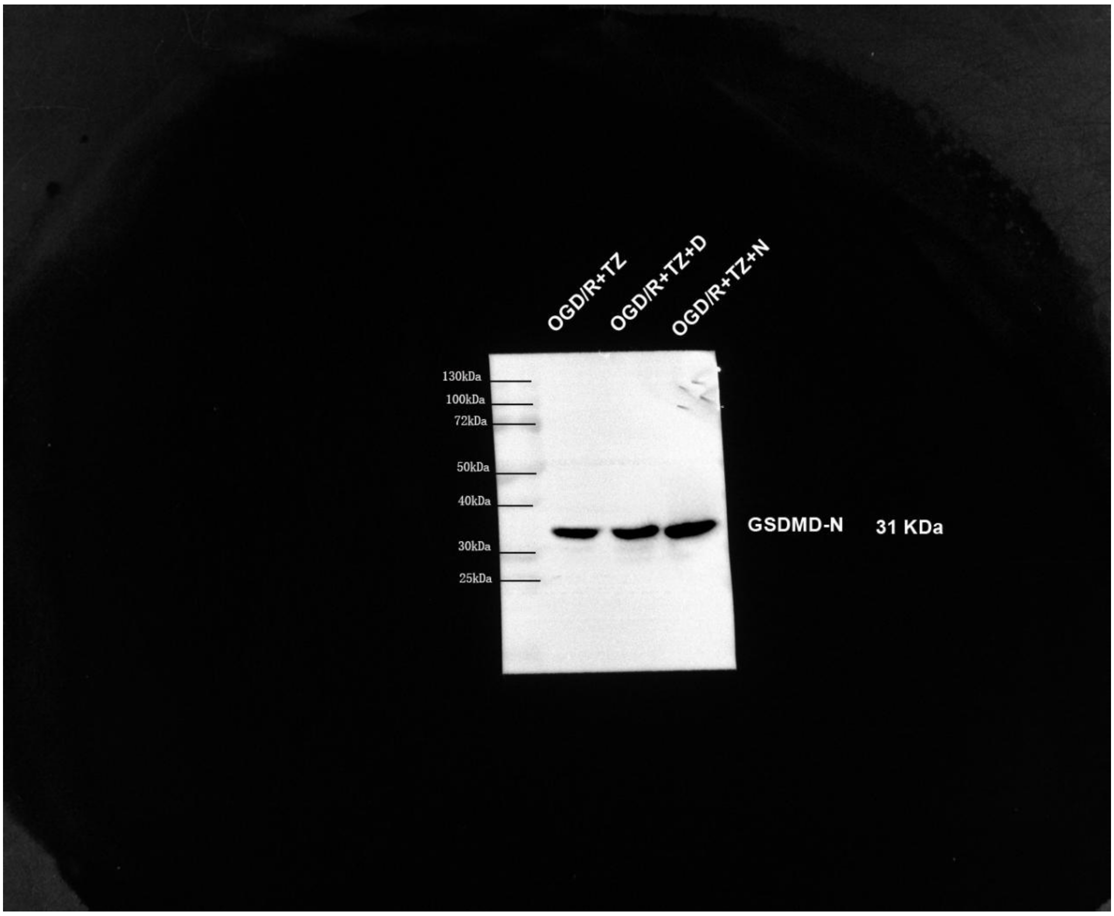
OGD/R+TZ
OGD/R+TZ+D
OGD/R+TZ+N
130kDa
100kDa
72kDa
50kDa
40kDa
30kDa
25kDa
GSDMD-N
31 KDa

6A-GSDMD-N

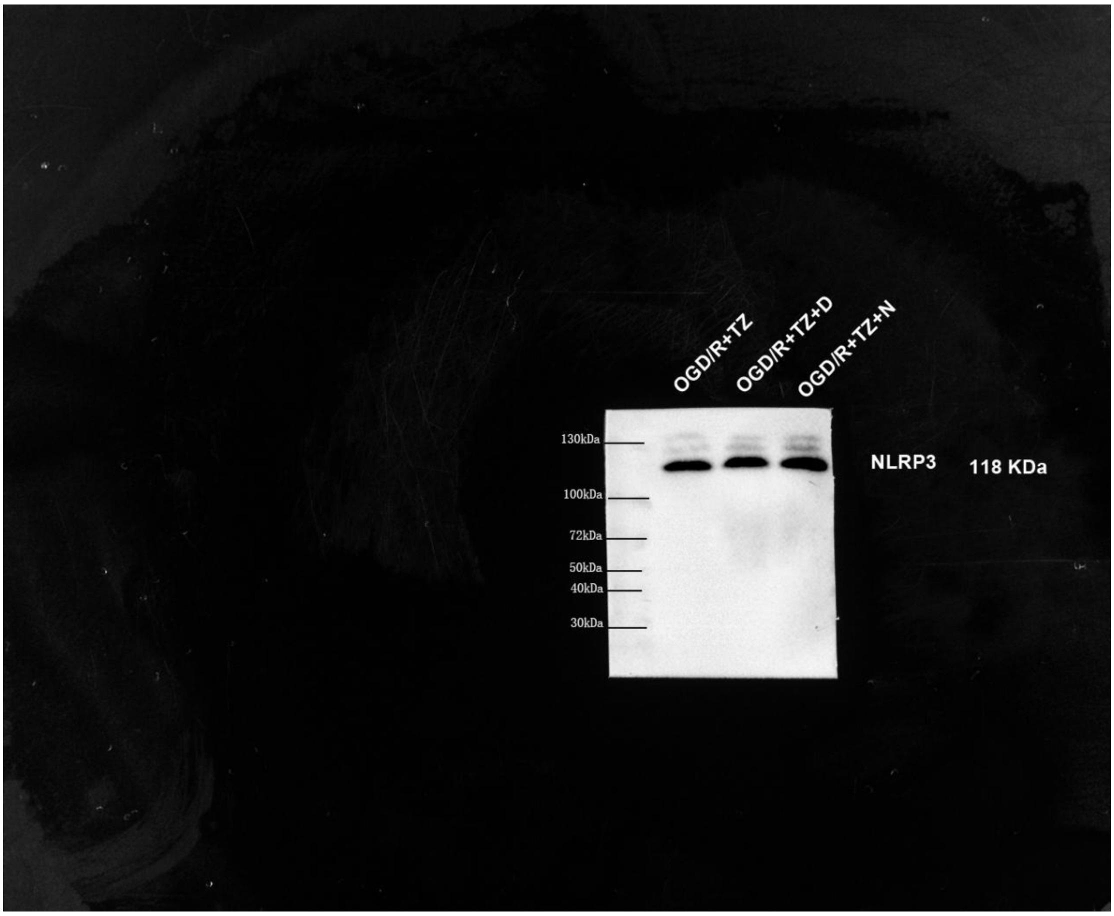
OGD/R+TZ
OGD/R+TZ+D
OGD/R+TZ+N
130kDa
100kDa
72kDa
50kDa
40kDa
30kDa
NLRP3
118 KDa

6A-NLRP3

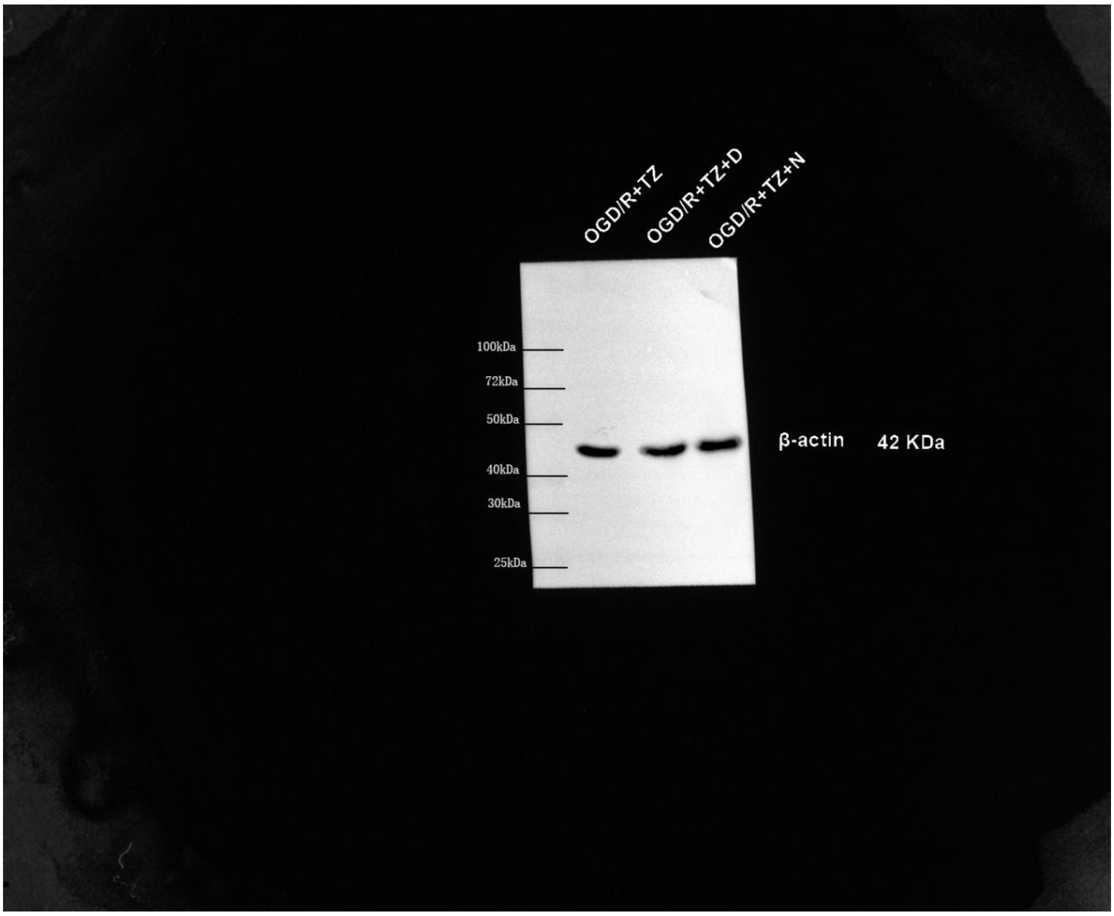
OGD/R+TZ
OGD/R+TZ+D
OGD/R+TZ+N
100kDa
72kDa
50kDa
40kDa
30kDa
25kDa
β-actin
42 KDa

6A-β-actin

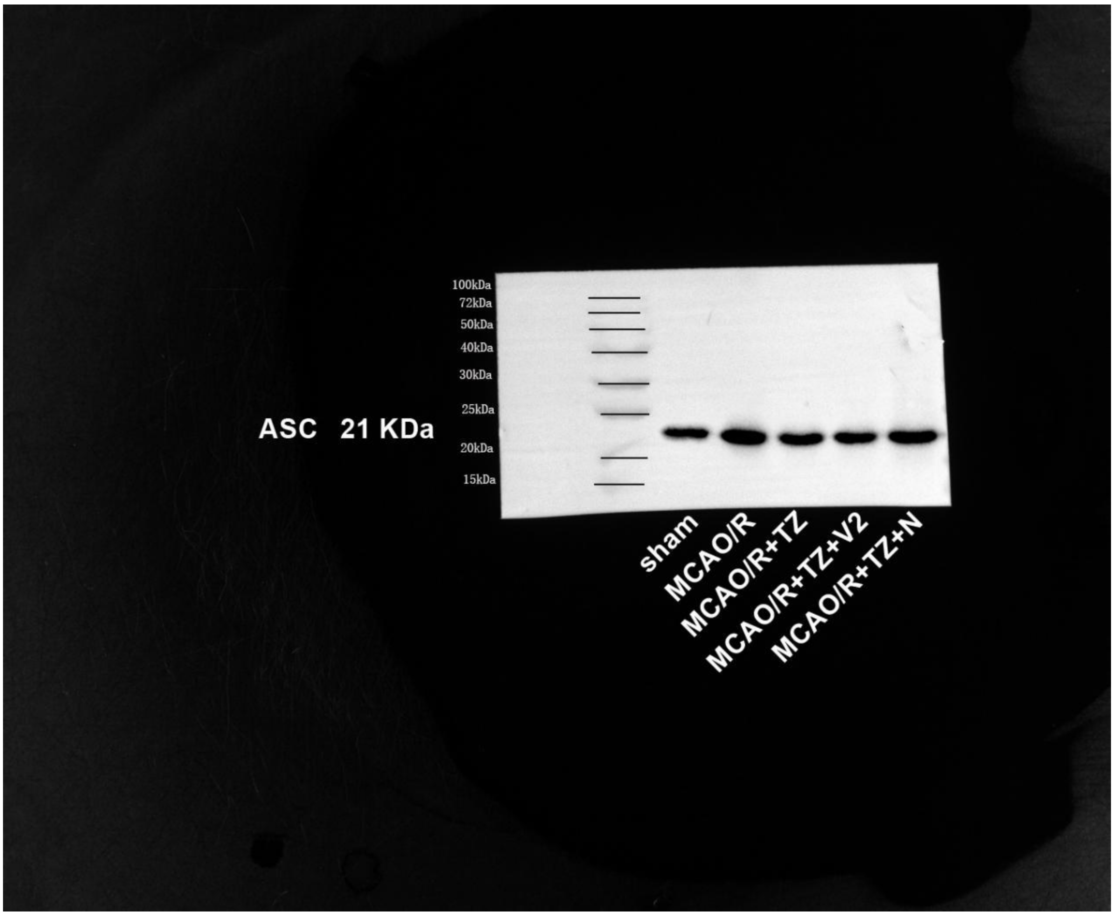

100kDa
72kDa
50kDa
40kDa
30kDa
25kDa
20kDa
15kDa
ASC 21 KDa
sham
MCAO/R
MCAO/R+TZ
MCAO/R+TZ+V2
MCAO/R+TZ+N

7B-ASC

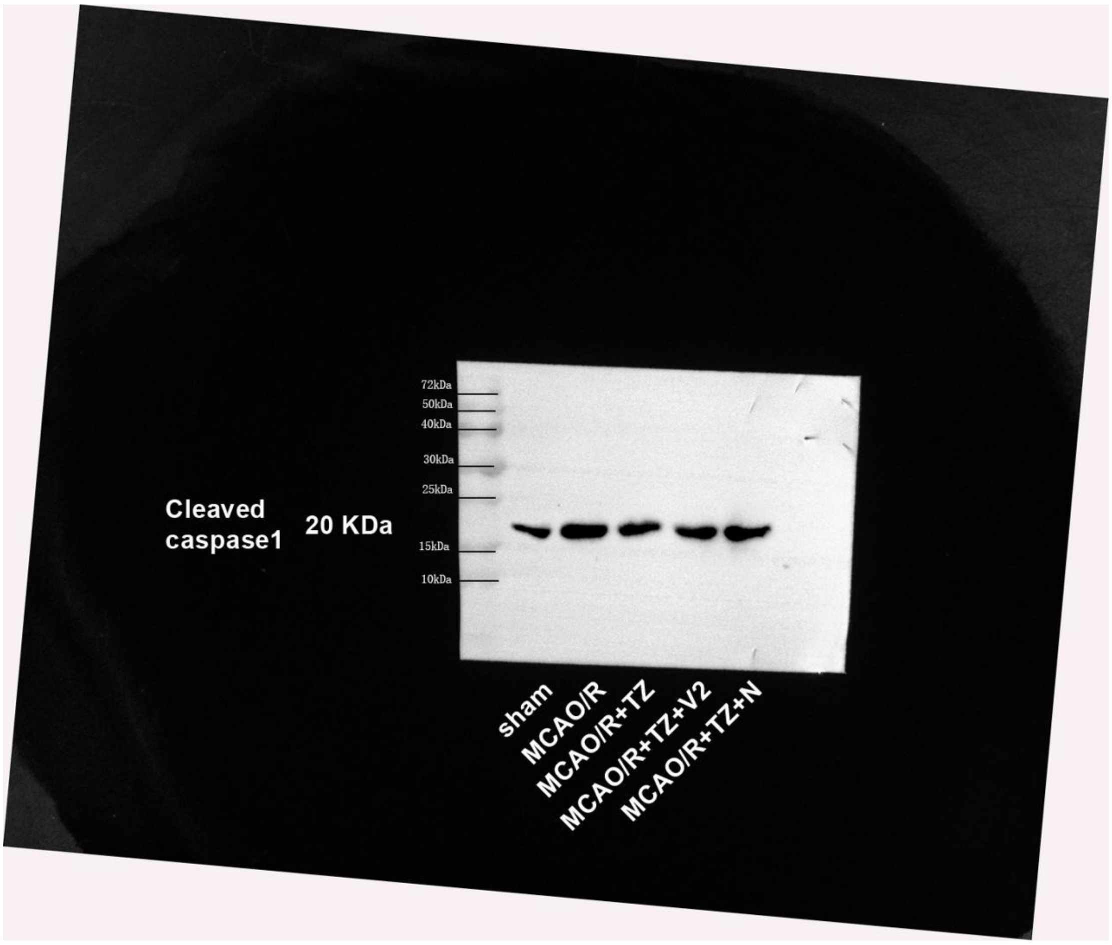

7B-Cleaved caspase1

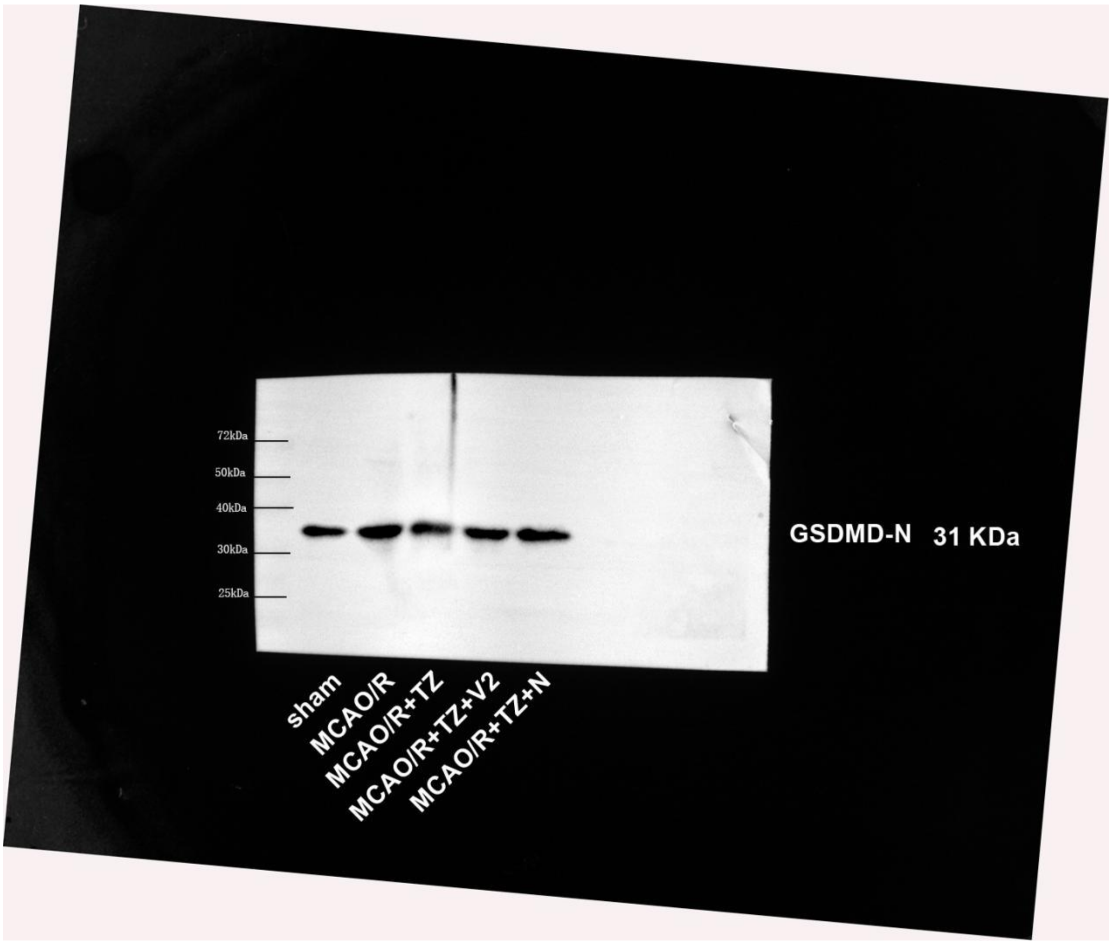
72kDa
50kDa
40kDa
30kDa
25kDa
GSDMD-N 31 KDa
sham
MCAO/R
MCAO/R+TZ
MCAO/R+TZ+V2
MCAO/R+TZ+N

7B-GSDMD-N

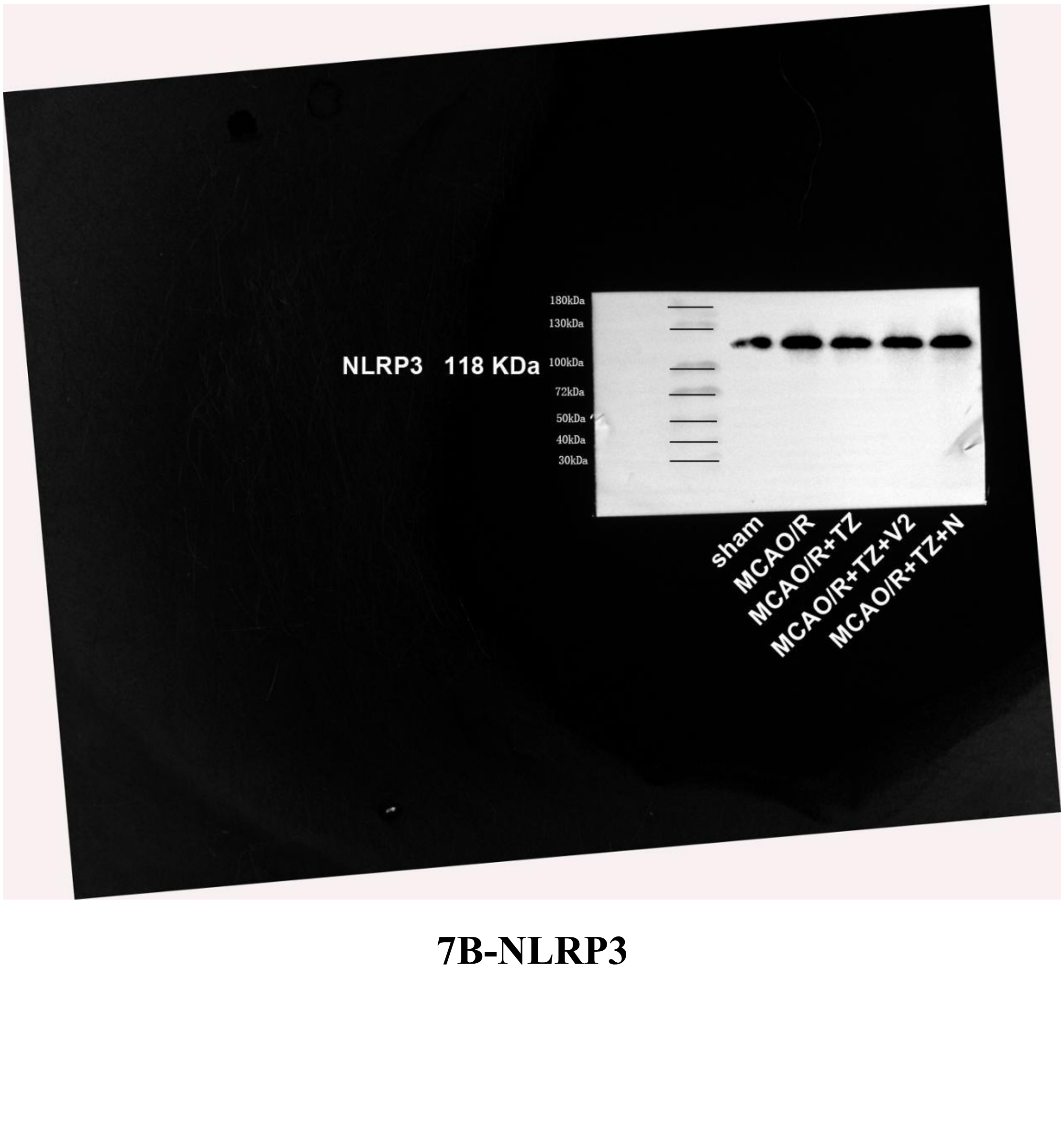
NLRP3 118 KDa
180kDa
130kDa
100kDa
72kDa
50kDa
40kDa
30kDa
sham
MCAO/R
MCAO/R+TZ
MCAO/R+TZ+V2
MCAO/R+TZ+N

7B-NLRP3

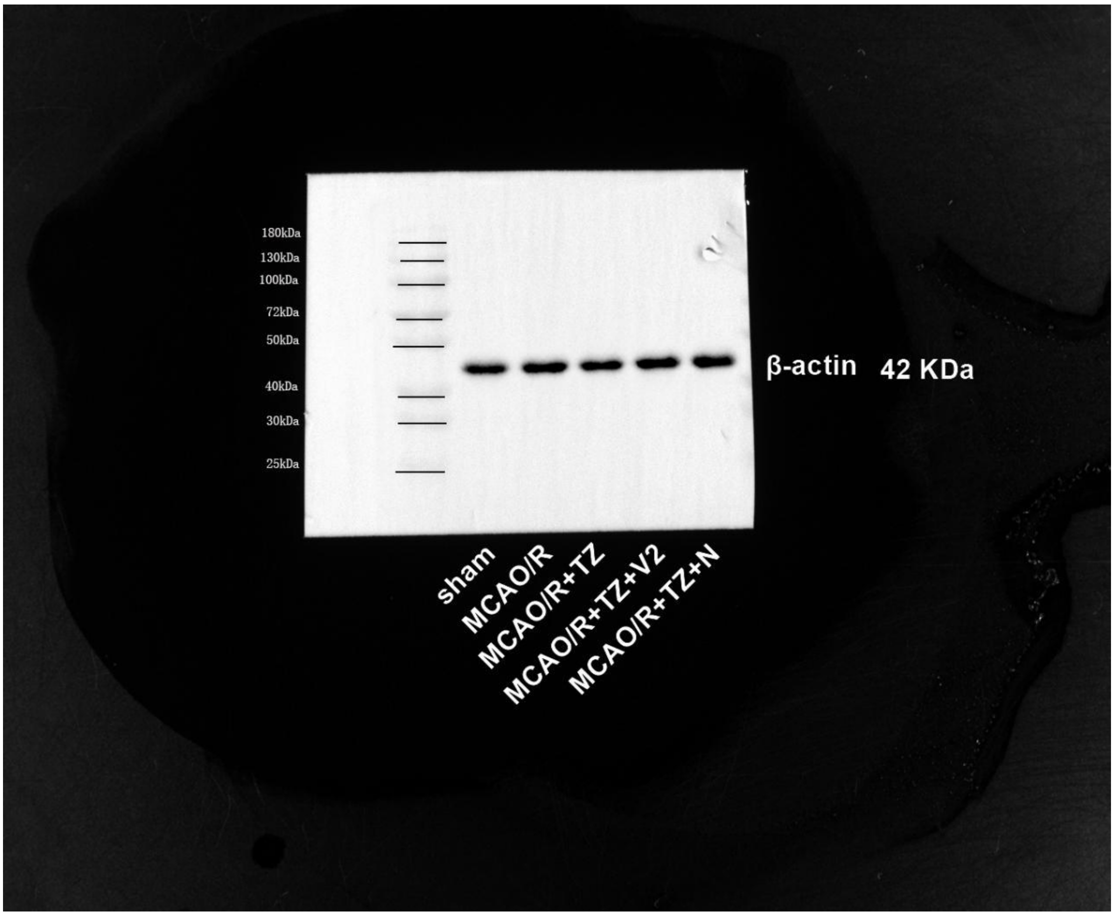
180kDa
130kDa
100kDa
72kDa
50kDa
40kDa
30kDa
25kDa
β-actin 42 KDa
sham
MCAO/R
MCAO/R+TZ
MCAO/R+TZ+V2
MCAO/R+TZ+N

7B-β-actin
